# Endothelial Dysfunction in Childhood Cancer Survivors: A Narrative Review

**DOI:** 10.3390/life12010045

**Published:** 2021-12-29

**Authors:** Marco Crocco, Giuseppe d’Annunzio, Alberto La Valle, Gianluca Piccolo, Decimo Silvio Chiarenza, Carolina Bigatti, Marta Molteni, Claudia Milanaccio, Maria Luisa Garrè, Natascia Di Iorgi, Mohamad Maghnie

**Affiliations:** 1 Neuro-Oncology Unit, Istituto di Ricovero e Cura a Carattere Scientifico Giannina Gaslini Institute, 16147 Genoa, Italy; giangi.piccolo@gmail.com (G.P.); Claudiamilanaccio@gaslini.org (C.M.); mluisagarre@gaslini.org (M.L.G.); 2 Department of Neuroscience, Rehabilitation, Ophthalmology, Genetics, Child and Maternal Health, University of Genova, 16100 Genoa, Italy; albertolavalle88@gmail.com (A.L.V.); silvio.chiarenza@yahoo.com (D.S.C.); carolina.bigatti.cb@gmail.com (C.B.); marta.molteni.1991@gmail.com (M.M.); natasciadiiorgi@gaslini.org (N.D.I.); mohamadmaghnie@gaslini.org (M.M.); 3 Department of Pediatrics, Istituto di Ricovero e Cura a Carattere Scientifico Giannina Gaslini Institute, 16147 Genoa, Italy; giuseppedannunzio@gaslini.org

**Keywords:** cancer survivors, endothelial dysfunction, noncommunicable diseases prevention, flow mediated dilation, carotid intima media thickness, peripheral artery tonometry, pulse wave velocity, atherosclerosis, vascular toxicity, cardiotoxicity

## Abstract

Assessment of endothelial dysfunction in cancer survivors may have a role in the early identification of non-communicable diseases and cardiovascular late effects. Oncological therapies may impair endothelial function. Therefore, in patients such as childhood cancer survivors who could benefit from early cardioprotective pharmacological interventions, it is essential to monitor endothelial function, even if the optimal methodology for investigating the multifaceted aspects of endothelial dysfunction is still under debate. Biochemical markers, as well as invasive and non-invasive tools with and without pharmacological stimuli have been studied. Human clinical studies that have examined lifestyle or cancer treatment protocols have yielded evidence showing the involvement of lipid and lipoprotein levels, glycemic control, blood pressure, adiposity, inflammation, and oxidative stress markers on the state of endothelial health and its role as an early indicator of cardiometabolic risk. However, with regards to pharmacological interventions, cautious interpretation of the result attained whilst monitoring the endothelial function is warranted due to methodological limitations and substantial heterogeneity of the results reported in the published studies. In this narrative review, an overview of evidence from human clinical trials examining the effects of cancer therapies on endothelial disease is provided together with a discussion of endothelial function assessment using the different non-invasive techniques available for researchers and clinicians, in recent years.

## 1. Introduction

Cancer and cardiovascular diseases are the most common causes of non-communicable diseases [1] and premature death in Western countries [2]. In recent years, improved survival rates in childhood cancer patients have increased the overall population of survivors. Recently, estimates have indicated that there will be over 12 million cancer survivors in Europe, including around 300 thousand childhood cancer survivors (CCS) [3]. The population of survivors is increasing over time, and with it has come greater recognition of the importance of the adverse effects of cancer therapies, the need for a better understanding of the etiopathogenetic mechanisms underlying cardiovascular damage after cancer therapies, and an improved ability to detect its first signs. CCS are significantly more likely to take medications for hypertension (odds ratio (OR), 1.9), dyslipidemia (OR, 1.6), or diabetes (OR, 1.7) than their sibling controls [4], and the risk of cardiovascular disease with premature mortality rate is 5–10 times more common [5]. Vascular toxicities are the second most common cause of death in long-term cancer survivors [6]. While clinical monitoring for cardiotoxicities has been described in numerous articles and validated by guidelines, only a few reports evaluating potential clinical strategies for monitoring vascular toxicity during and following anticancer treatment exist, reflecting a serious gap in our current knowledge and the need to identify potential non-invasive methods to assess vascular toxicity [7].

A normal endothelium assures antiplatelet, anticoagulant, and anti-inflammatory actions. Endothelial impairment may be the first step of vascular toxicity and is the primum movens in the pathogenesis of atherosclerosis and thrombosis which leads to cardiovascular diseases (coronary heart disease, hemorrhagic or ischemic stroke, peripheral arterial disease, and venous thromboembolism) [8]. Cancers and oncological therapies may damage the delicate endothelial cell system, which supports the balance between vasodilating and vasoconstricting substances produced by (and acting on) endothelial cells. The reaction against hemodynamic stress on the damaged endothelium causes thrombosis by producing plasminogen activator inhibitor which reduces the generation of plasmin by inhibiting tissue plasminogen activators and urokinase [9]. Furthermore, the desquamation of endothelial cells exposes the von Willebrand factor (vWF), a stimulus for platelet activation and aggregation, at the level of the subendothelial basement membrane. The inflammation may cause a procoagulant state through an increase in adhesion molecules (E-selectin, vascular cell adhesion molecule, and intercellular adhesion molecule), vasoconstrictor agents (endothelin-1 (ET-1) and tissue factor), chemokines, and proinflammatory cytokines (interleukin-1, interleukin-6, interleukin-8, and interferon gamma) [10]. A link between prothrombotic and inflammation state has been described and termed “immuno-thrombosis”. This relationship is bidirectional, with the release of inflammatory mediators activating the endothelium towards a procoagulant and platelet activating phenotype. The generation of procoagulant agents and tissue factors cause vasculitis and indeed, a proinflammatory state [11].

Among the long-term complications reported in CCS, metabolic syndrome (MS) and its consequences deserve attention [12]. The term MS was extensively described by Reaven et al. [13], who indicated a cluster of clinical signs and symptoms which included central obesity, insulin resistance, high blood pressure levels, high levels of triglycerides, low levels of high-density lipoprotein (HDL), and different degrees of dysglycemia [14]. The prevalence of MS in the pediatric population is increasing worldwide, mainly linked to epidemic obesity. MS represents a cardiometabolic risk factor for the development of atherosclerosis, cardiovascular disease, and type 2 diabetes mellitus [15]. In adolescents, MS seriously impairs global health and also the quality of life. At present, a valid, globally accepted definition of MS is lacking, and more than 46 different definitions have been proposed, mainly based on an adult classification [16]. In 2007, the International Diabetes Federation established a new set of diagnostic criteria [17]. Therefore, a correct diagnosis of MS is sometimes difficult, since age- and gender-specific parameters (i.e., blood pressure level percentiles, insulin resistance indexes, lipid profile, and body mass index (BMI) are different from those applied to adults.

The pathogenesis of MS is still unclear, and different mechanisms have been hypothesized. Insulin resistance together with central and visceral obesity trigger various pathways that result in a proinflammatory and prothrombotic state leading to endothelial damage. Visceral fat acting as an endocrine organ rather than subcutaneous adipose tissue plays a pathogenetic role in MS. In fact, adipocytes secrete several inflammatory markers and adipokines involved in energy expenditure, endothelial metabolism, and atherogenesis [18]. MS in CCS was first noted in 1996 [19] and several studies linked cardiovascular risk factors to MS. It has been reported that CCS, in particular, with acute lymphoblastic leukemia (ALL), tended to become obese, and to develop MS [20]. Several pathogenetic mechanisms underlying MS have been considered to play important roles in CCS: obesity, dyslipidemia, genetic factors, in particular leptin receptor polymorphism, and treatments for childhood cancer [21,22]. Surgery, especially brain surgery, impairs the hypothalamus–pituitary axis, with subsequent hormonal deficiencies. Moreover, gonadectomy and thyroidectomy may represent a risk factor for MS [23]. Radiotherapy could cause gonadotropin, adrenocorticotropin, and thyrotropin axes impairment [24], and in case of high dosages, the neurocognitive function could also be damaged, with consequent impairment of cell metabolism and reduced physical activity. Different organs may be directly damaged by local radiation [25]. In particular, cranial radiotherapy induces growth hormone deficiency, linked to MS and endothelial dysfunction [26]. Hypothalamic–pituitary axis impairment due to cranial radiotherapy may impair energy expenditure by hypothalamic resistance to leptin negative feedback, with subsequent obesity. Cancer survivors are globally less physically active than healthy peers [27,28]. Endothelial damage, which includes carotid and femoral intima media thickness, has been reported in CCS who underwent neck and chest radiotherapy. Chemotherapy impairs endocrine function, and several agents disrupt DNA replication and transcription with the subsequent impairment of cell growth and repair; platinum, alkylating agents, and anthracyclines (AAs) produce reactive oxygen species (ROS) leading to mitochondrial dysfunctions. Moreover, specific side effects of chemotherapy, including, cellular lysis, apoptosis, and anemia cause the production of inflammatory cytokines and macrophage activation involved in MS and endothelial damage pathogenesis [29].

Due to the increasing number of cancer survivors with elevated cardiovascular risk and the need for long term follow-up, there is a clear need to evaluate substitute methods for the current invasive assessment of cardiovascular damage. The stratification of cardiovascular risk in these patient groups requires the use of validated, reproducible, and easily available methods to evaluate endothelial dysfunction. In recent years, experience with the use of non-invasive methods to evaluate endothelial function in children has been increasing proportionately to technological advancements. Some of these non-invasive methods are already available for routine clinical practice. The most promising non-invasive tests used are ultrasonographic markers as well as arterial stiffness, peripheral arterial tonometry, and circulating blood markers; although, for many of them, reference values are still under investigation, making the interpretation of their results in the pediatric context difficult [30].

## 2. Endothelial Dysfunction in Childhood Cancer Survivors

In this review, we aim to discuss experimental studies that should provide insight into the pathophysiological mechanisms of endothelial toxicity after cancer therapies, and the interconnection of these mechanisms with metabolic risk factors, which might also lead to an improved understanding of cardiovascular diseases in cancer survivors. This review also provides a detailed description of the methodology, limitations, and current pediatric experiences, in recent years, associated with the most used non-invasive methods to evaluate the endothelial function. Finally, one of the main aims of this review is to discuss oncological and metabolic risk factors for endothelial dysfunction in CCS.

The authors M.C., A.L.V., and G.D. independently and systematically searched the MEDLINE/Pubmed database (United States National Library of Medicine National Institutes of Health) up until 1 June 2021. The research was carried out with the following PubMed MeSH terms in order to select the existing data in the literature: ((child* [Title/Abstract] Or infant* [Title/Abstract] OR adolescent* [Title/Abstract] OR children [Title/Abstract] OR pediatric* [Title/Abstract] OR childhood [Title/Abstract])) AND (arterial dysfunction [Title/Abstract] OR arterial function* [Title/Abstract] OR arterial stiffness [Title/Abstract] OR endothelium dysfunction* [Title/Abstract] OR endothelial dysfunction* [Title/Abstract] OR endothelial impairment [Title/Abstract] OR endothelial function* [Title/Abstract]) AND (tumor* OR tumour* OR neoplasm OR maligna* OR leukemia OR leukaemia OR oncology OR brain tumour OR cancer*).

The research was extended to studies published in the last two decades (2000–2021).

### 2.1. Assessment of Endothelial Function in Pediatric Age

#### 2.1.1. Peripheral Arterial Tonometry

The reactive hyperemia index (RHI) by Endo-PAT 2000 is a non-invasive method to assess endothelial function by measuring modifications in digital pulse volume during reactive hyperemia, and represents a non-invasive, reproducible, and operator-independent tool that can detect precocious endothelial dysfunction [31].

Endo-PAT 2000 evaluates microvascular endothelial function. The technique provides values for the calculation of an RHI peripheral artery tonometry (PAT), which is generated by brachial artery blood flow occlusion for 5 min, through rapidly inflating a blood pressure cuff to a suprasystolic pressure of 60 mmHg above the patient’s systolic pressure or 200 mmHg. The PAT signal is measured by recording finger arterial pulsatile volume changes through plethysmographic biosensors that impart a uniform sub-diastolic pressure field to the distal two-thirds of the fingers. The RHI is calculated automatically from the differences between post- and pre-occlusion PAT signal ratio in the occluded arm, relative to the same ratio in the control arm, and corrected for baseline vascular tone. In adults, the RHI correlates with the measurement of endothelial vasodilator function in the coronary arteries, cardiovascular risk [32,33,34], and brachial flow-mediated dilation (FMD) [35].

To date, control groups are needed due to a lack of reference values in the pediatric population. In addition, the variable factors, mainly pubertal development, need adjustments to the reference values according to the Tanner stages. Related to this purpose, Bhangoo et al. reported that enhancement of the PAT index was positively related to the Tanner stage, in 89 healthy school-age boys and girls [36]. The data were confirmed in a subsequent study of 94 healthy children and adolescents [37]. The main factors involved seem to be the sex steroid hormones. In addition, Endo-PAT 2000 showed excellent reproducibility and feasibility in a population of 30 healthy adolescents (aged from 13 to 19 years) evaluated on two different days, separated by no more than seven days [38].

#### 2.1.2. Ultrasonographic Markers

The measurement of FMD is the most common method used for evaluating endothelial vasodilating function. The evaluation of FMD is based on measuring relative arterial dilation in downstream arteries in response to ischemia induced by cuff inflation. The European Society of Hypertension (ESH) recommends measurements of FMD below the elbow [9].

It must also be stressed that training for an FMD operator is required and results may be invalidated by observer variability. Due to the presence of these limitations, in pediatric populations, reference ranges have been proposed by several authors [39,40], but not without some discrepancies between different publications. In fact, it is very difficult to establish reference ranges, especially in children, where arterial size is difficult to measure and influences FMD results (smaller arteries are associated with a greater FMD and baseline size should be considered as a covariate in the analysis of the results). Indeed, experts from the American Heart Association (AHA) recommend comparisons with a control group in all studies in children until better pediatric reference ranges are available [41].

In pediatric populations, low FMD values were found in several chronic diseases associated with high cardiovascular risk: chronic kidney disease [42], nephrotic syndrome [43], diabetes type 1 [44], and obesity [45,46]. FMD is a measurement of macrovascular endothelial health.

Measurement of carotid intima-media thickness (cIMT) can be used as a marker of structural changes in large arteries. It can be used to quantify prodromal stages of atherosclerotic lesions and to monitor them changing over time [47]. An increase in cIMT is believed to reflect a compensatory adaptation of intimal and medial layers to changes in pressure and flow that precede atherosclerotic lesions [48].

Recommendations for measurement of cIMT in pediatric patients have been published by the AHA [41], and more recently, also by the Association for European Paediatric Cardiology (AEPC) [49]. The measurement should be performed in both carotid arteries with an ultrasound system using high-resolution (>7 MHz) broadband linear probes that allow for digital image acquisition, storage, and review. The measurement is usually performed within the distal wall of the common carotid artery, most commonly 10 mm below the carotid bulb and should be performed in the end-diastolic phase. Electrocardiogram (ECG) or other cardiac cycle tracking methods are needed. An analysis of the digitally stored loops should be performed preferably on a high-resolution monitor using a validated dedicated software [49].

The ESH [50] and AEPC [49] guidelines recommend using normative values published by Doyon et al. [47]. These reference charts were sex-specific normalized to age or height and constructed from 1051 non-obese and non-hypertensive children aged 6 to 18 years. The authors highlighted that cIMT showed a positive correlation with age, height, BMI, and BP. A significant sex difference was apparent from the age of 15 years.

Measurement of IMT is also possible in other sites; the most frequently used sites are the femoral artery (fIMT) and abdominal aorta (aaIMT). Evaluation of aaIMT is an interesting option due to the fact that atherosclerosis first develops in the distal aorta (and coronary arteries) and an abdominal ultrasound is easy and well tolerated in infants. In the pediatric age, aaIMT has been shown to be positively related to triglyceride levels, systolic and diastolic blood pressure (SBP and DBP), BMI, waist-to-hip ratio, diabetes mellitus, and premature birth [51].

Even if the long-term benefit of IMT measurement on a single patient’s vascular health remains to be determined, the AEPC Working Group on Cardiovascular Prevention strongly recommends the use of cIMT for screening patients with elevated cardiovascular risk. The authors conclude that the cIMT measurement offers, in addition to the conventional cardiovascular risk factors screening, direct, fast, easy to apply, and reproducible information about the vascular status of pediatric patients [49].

However, currently, there has been no consensus on the part of scientific associations about the clinical indications of IMT measurement in childhood diseases. For example, the American National High Blood Pressure Education Program Working Group [52] and European guidelines [50] for high blood pressure in children and adolescents, do not recommend the routine evaluation of IMT in hypertensive children.

#### 2.1.3. Arterial Stiffness

Arterial stiffness has been recognized as an effective early indicator of cardiovascular risk [53]. With aging, the loss of elasticity of the arteries, due to the replacement of elastin with collagen fibers, may initiate an atherosclerotic lesion. Arterial stiffness is a dynamic parameter that depends on both the vascular structure and function, particularly due to the elastic properties of the arterial tree. The energy of pulsatile blood flow generated by cardiac contraction is absorbed into the large arterial elastic walls, and then converted into laminar flow that goes through the small arteries and capillaries. In children, elastic properties of the arteries are mostly dependent on age and height [54]. Arterial elastic properties depend mainly on the presence of elastic fibers in the vessel wall, which have a maximum rate in the perinatal period followed by a fast decrease already during childhood [55]. Arterial stiffness may be evaluated using several non-invasive methods that can be classified based on the operating mechanism into three models: transmission or propagation model (e.g., various pulse wave velocity (PWV) measurements and pulse wave analysis (PWA)); pulsation or distension model (e.g., ultrasound-derived carotid artery compliance and distensibility, ambulatory arterial stiffness index (AASI)); Windkessel model (e.g., systemic arterial compliance via area method) [56].

An evaluation of arterial stiffness is economical, easy, and reliable due to several techniques developed to assess the elasticity of the blood vessel. Non-invasive methods to assess arterial stiffness include PWV measurements, PWA, measurements of the augmentation index (AI), arterial distensibility, and AASI based on 24 h ambulatory blood pressure monitoring (ABPM). Many of these devices are now available for research and clinical use [30].

- Pulse Wave Velocity

PWV is a simple, non-invasive measurement of the arterial stiffness utilizing ultrasound or other methods, such as applanation tonometry, to analyze wave forms. A pulse wave generated by contraction of the left ventricular propagates along the arterial tree, at a velocity that depends on the geometric and elastic arterial properties. The method of choice for assessing PWV is applanation tonometry by evaluating the pulse wave in the proximal (carotid artery) and distal artery (femoral artery) simultaneously with an ECG. The distance from the carotid artery pulse to the sternal notch and the distance from the sternal notch to the femoral artery pulse should be measured for each patient. Then, the tonometers are placed on the proximal and distal arteries to obtain arterial waveforms synchronized to the R-wave. A similar approach for measuring PWV is oscillometry using two cuffs placed on the arm and the ankle, but in contrast to applanation tonometry, the oscillometric measurements are user independent [57].

The PWV reference values for children, for three different devices, were included in the ESH 2016 Guidelines [50] and published in three studies. Between 2006 and 2009, Reusz et al. used the applanation tonometry method with a PulsePen device (DiaTecne SRL, Milan, Italy) to assess carotid femoral PWV (PWVcf) in 1008 healthy children and young adults (450 Hungarian, 455 Italian, and 103 Algerian), aged 6–20 years [58]. The applanation tonometry method was also used by Thurn et al. with a Vicorder device, (SMT Medical GmbH&Co., Wuerzburg, Germany) to assess PWVcf in 1003 children and adolescents (713 German and 390 Turkish) [59]. In addition, between 2011 and 2013, Elmenhorst et al. estimated PWV by oscillometric blood pressure measurement with a Mobil-O-Graph device (IEM GmbH, Stolberg, Germany) in 1445 healthy German children and young adults aged 8–22 years [60]. The adequacy and reliability of the Mobil-O-Graph device seems to be insufficient for clinical use as compared with other different non-invasive devices or with the invasive measurement of aortic PWV (PWVao) as demonstrated by Salvi in two recent studies [61,62]. The reference range in the pediatric population was recently updated and revised by Hidvégi et al. (2021) [63]. They took into consideration the remarkably changed BMI and SBP/DBP cut-off values that occurred in this population during the last decade, and measured PWVao using an occlusive-oscillometric device (Arteriograph, TensioMed Ltd., Budapest, Hungary) in a healthy population of 4690 (2599 boys) children aged 3–18 years. Different mean values were found as compared with a previous publication (Hidvégi et al. (2012) [64]), and the mean PWVao values were significantly lower in this new study (in boys aged 9–16 and in girls aged 11–17). The differences in reference values of PWV measured by various authors may also be influenced by the different measurements applied. The limitation is evident when the PWV values from cardiovascular magnetic resonance (CMR) and applanation tonometry are compared [65]. The assessment of PWVao showed good agreement with PWV measurements obtained from invasive pressure measurements as the gold standard, but longitudinal studies in the USA/Europe are lacking [66]. Magnetic resonance imaging (MRI) can be applied in much the same way as ultrasound to determine PWV or arterial distensibility. When used to assess PWV, an MRI has the advantage of measuring the distance between, and the area of, the arteries more accurately [67]. It can evaluate the aortic distensibility much better than an ultrasound, however it is a methodology that requires a lot of time, high costs, and trained staff.

- Pulse Wave Analysis

A PWA measures the arterial pulse wave; currently, there are several methods available (for example, tonometry, oscillometry, and plethysmography) but, usually, the applanation tonometry is the most commonly used method [68]. An arterial pulse wave is generated by the sum of the propagating wave (initiated by a left ventricular contraction) and the returning wave (reflected from peripheral vessels). The aortic pulse wave parameters may be estimated using a mathematical computer analysis of the input data (radial artery pulse wave and a simple brachial blood pressure). A primary outcome derived from PWA is the AI, which is the ratio between the systolic peak and the first systolic inflection of an arterial pulse wave, corrected for peripheral pulse pressure and for beats per minute. AI is an indirect measure which is an expression of the reflected wave coming from the periphery to the heart and is based on the principle that an increase in the stiffness is associated with faster propagation of the forward pulse wave as well as an earlier return of the reflected wave. The AI may be used to assess arterial stiffness.

- Arterial Distensibility and Other Methods

Another method to assess arterial stiffness is the measurement of arterial distensibility, which is a condition of the arterial wall that is the converse of arterial stiffness [56]. It is a parameter that measures (usually in the aortic carotid, brachial, radial, or femoral arteries) the change in the transverse arterial dimension induced by blood pulse pressure. Distensibility may be measured by cross-sectional views with ultrasound or by radiofrequency echo tracking. The measurement of arterial distensibility using radiofrequency waves does not required trained personnel and excludes observer error. Recently, Voges et al. updated the normal range of aortic distensibility by CMR in 71 children and young adults aged 2.3–28.3 years [67].

The AASI is an indirect measure of arterial stiffness calculated as 1 minus the regression slope of the DBP to SBP over a 24 h ABPM. The AASI has the advantage over other measures of arterial stiffness due to its low cost and full automation [69].

Except for PWV, the other methods used to assess arterial elasticity are currently burdened by important limitations that interfere with pediatric clinical use. In particular, normative values are not available for younger patients, the minimum age of the normative reference values are 6 years old (and a height ≥ 120 cm) for arterial distensibility and 5 years (and height ≥ 120 cm) for ABPM-derived techniques [70], and only for the AI or arterial distensibility the reference values are available from 3 years [63]. Measurement of the PWA is highly dependent on the precise acquisition of the signal, and therefore patient cooperation may limit the use in younger children. Furthermore, even if a patient is cooperative (also in younger adolescents) it can be difficult to attain a sufficiently strong signal from small arteries.

While PWV measurements and a PWA can be considered as “direct measurements” of arterial stiffness, the ABPM-derived indices and arterial distensibility are commonly described as “indirect measurements”. The AHA recommended that (Class I, level of evidence A) arterial stiffness should be determined non-invasively by the measurement of PWVcf [71].

#### 2.1.4. Circulating Blood Markers

Several circulating markers of endothelial dysfunction have been studied over the years. The search for a blood marker was stressed by the usefulness of defining an indicator of cardiovascular risk that is easily assessable and repeatable over time. Studies have focused on vascular adhesion molecules [72], coagulation proteins [73], nitric oxide metabolites [74], proteins involved in calcium phosphate deposition in the arterial wall [75], circulating endothelial progenitor cells [73], as well as cytokines and other proinflammatory molecules involved in endothelial damage as the first act of the atherosclerotic process [76,77].

Although circulating markers of endothelial dysfunction represent a promising field of study, based on the available evidence, clinical use of any of these markers cannot be recommended yet due to currently low reliability of the tests used and the absence of established reference values for any of the soluble markers of endothelial dysfunction.

### 2.2. Assessment of Endothelial Dysfunction in Childhood Cancer Survivors

**Figure 1:**
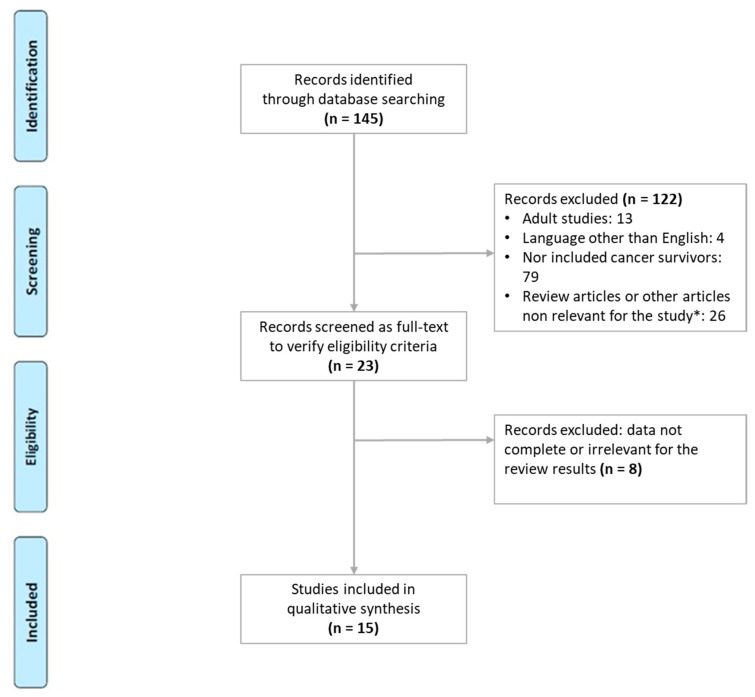
Flow chart for eligible studies. * letters to editors, book chapters, study protocols, case reports.

A total of 145 citations were found in MEDLINE/PubMed; 122 records were excluded because they were not written in English, did not include cancer survivors, or the cancer survivors were over the age of 18. Therefore, 23 full texts were assessed for eligibility, of which 15 pediatric papers from 2000 to 2021, were considered (Figure 1).

The authors also evaluated the possible presence of additional studies by searching letters to editors, book chapters, study protocols, case reports, the references of the primary studies and review articles, and did not finding additional clinical studies which were eligible for review.

Table 1 and Table 2 summarize the endothelial dysfunction results, as well as cancer and metabolic characteristics of the studies.

In a study on circulating blood markers, Okur et al. showed that the level of integrin and selectin in CCS with solid tumors, treated with AAs, did not differ as compared with a healthy control (HC) group. While the FMD values of the patients with a cumulative AA dose ≥ 300 mg/m^2^ were significantly lower than those of the patients with lower cumulative AA dose and HCs, a significant negative correlation between FMD and increasing cumulative AA dose were found (r = −0.287). In the same study, Okur et al. also found a significant difference between the mean cIMT of the CCS and healthy children [87].

FMD has been successfully used as an indicator of endothelial dysfunction by other authors. Chow et al. demonstrated a significant decrease in FMD measurement among 14 patients with cancer, after 2–60 months of treatment with a dose of more than 300 mg/m^2^ cumulative AA [79]. Further, Jang et al. used FMD to evaluate vascular endothelial function in 21 children with ALL who were treated, 2–85 months before the evaluation, with a lower dose of AA chemotherapy (the cumulative dose of AAs was 142.5 ± 18.2 mg/m^2^) [82]. The authors found that FMD was significantly lower in the patients as compared with a control group, whereas the time elapsed after the last AA treatment and the age at the time of treatment did not affect the change in FMD. In both studies, FMD in the brachial artery was used as the only method for the assessment of endothelial dysfunction, and it was still not clear that AAs induce vascular endothelial damage by rapid-onset process or by a mechanism which needs time such as apoptosis. In a study by Jenei et al., both the cumulative AA dose and the age at the start of treatment were found to be associated (independently) with FMD, distensibility, and stiffness index. Long-term survivors of childhood cancer who received AAs had poorer endothelial function and aortic stiffness as compared with both those of age- and sex-matched healthy individuals who did not receive cancer therapy, and those of age- and sex-matched survivors treated with chemotherapy without AA. A decrease in FMD % and an increase in aortic stiffness persisted long (more than 10 years) after AA treatment. However, the difference in FMD % disappeared in nitrate-mediated dilatation % ((NTG %) not significantly different among three groups), probably indicating that a decreased FMD % response was not due to smooth muscle dysfunction but purely due to endothelial cell dysfunction [83].

Discrepancies in cardiovascular outcomes from different studies were evident by comparing studies that used PWV measurements as a method. Herceg-Cavrak et al. evaluated PWVao using the oscillometric method (Arteriograph TensioMed device) in 53 children and adolescents (aged 6–20 years) treated with AA at least a year before, and in a control group of 45 age- and sex-matched healthy children. The PWVao significantly increased in patients treated with AAs resulting in increased arterial stiffness. There was no correlation between PWVao and the dose of AAS, and no difference was found in the blood pressure between HC and CCS [81]. Chaosuwannaki et al. examined the PWV using CMR measures in 40 adult patients undergoing AA chemotherapy and 13 age- and sex-matched controls. In the controls, PWV remained stable at the baseline and at follow-up 4 months later, while in cancer patients, PWV was significantly increased at the four-month follow-up visit. After adjusting for age, sex, and various clinical factors (BMI, SBP, HR, pulse pressure, serum hemoglobin, hypertension, diabetes, hyperlipidemia, resting cardiac output, and cardioactive medications) the difference in PWV between cancer and control patients at the four-month visit remained significant [94]. Krystal et al. confirmed that CCS older than 18 years had significantly higher PWV than controls in the same age group, which remained true when adjusted for age, sex, and BMI z-score [86]. As compared with the PWV norms established for healthy adults, 70% of CCS older than 18 years had an elevated PWV [95], suggesting that this group was at a higher risk of cardiac morbidity and mortality. However, the same study showed that CCS and HCs had similar PWV overall. No decisive change was observed in PWV in subsequent studies [90,92]. In a cross-sectional study involving 40 CCS (6–18 years, mixed cancer entities), Keiser et al. found an increase in peripheral SBP and DBP, as well as central SBP values as compared with the national reference values for healthy children and adolescents [92]. The PWV was elevated (PWV were assessed using the Mobil-O-Graph), but not significantly as compared with the reference values from Elmenhorst et al. [60] (<8 years: z-score 1.15 ± 2.89, *p* = 0.374 and ≥8 years: z-score 0.55 ± 1.90, *p* = 0.127). This result confirmed a previous study by von Korn et al. [90]. However, the different methods and measurement tools used in the various studies, as well as their manufacturers [81,86], did not allow a comparison of the PWV results. Moreover, Keiser et al. did not find any associations between increased central blood pressure or PWV and AA cumulative dose [92]. This could potentially be due to a shorter post-treatment period as compared with previous studies [83,94].

Several authors have used cIMT to evaluate endothelial dysfunction in CCS. Dengel et al. assessed carotid artery stiffness (compliance and distensibility), cIMT, brachial artery endothelial-dependent dilation, and endothelial-independent dilation using ultrasound in 319 CCS (participants were 9–18 years of age at examination) who were more than 5 years from the primary diagnosis and 208 siblings who had never been diagnosed with cancer [85]. CCS with tumors of the central nervous system (CNS) or leukemia had higher body fat percentage than controls, without any difference in cIMT, weight, and BMI. Leukemia CCS had lower measures of vascular function in both the carotid and brachial arteries. Moreover, these patients showed reduced carotid compliance and distensibility indicating increased arterial stiffness. Although CNS CCS did not have the same level of vascular dysfunction as survivors of leukemia the structure of the carotid showed significant differences due to thicker cIMT in CNS CCS. Carotid IMT did not differ between leukemia and non-CNS solid tumor survivors or controls. Nevertheless, it should be noted that the mean difference of 0.02 mm was equal to the estimated annual progression of cIMT (0.02–0.05 mm) and atherosclerosis [96]. In addition, Sherief et al. highlighted the difference in cIMT between childhood ALL survivors and HCs [93]. Similar results were observed by Okur et al. in solid tumors treated with AAs [87] and Muggeo et al. in ALL survivors with 25-OHD deficiency [89].

Few studies have analyzed endothelial dysfunction by RHI-PAT in CCS [97]. Masopustová et al. [88] evaluated the endothelial dysfunction in pediatric ALL survivors with biochemical markers and RHI-PAT. The results of the study showed that a combined approach may be used for the detection of endothelial dysfunction in ALL survivors. Blair et al. examined the endothelial function in survivors of solid tumors or hematopoietic malignancy treated with chemotherapies (AAs or platinum agents or antimetabolites) and/or radiation, who have been off therapy for more than three years. They showed a low/borderline RHI-PAT value without a measurable change in vascular function after four weeks of supplementing meals with flavonoid-rich purple grape juice [84].

In recent years, multiple studies have been conducted on serum endothelial markers as indicators of endothelial dysfunction [98,99], due to the simplicity of execution and applicability (with low invasiveness) in patients who are subject to routine blood sampling for monitoring the cancers and/or the side effects of therapies.

Luzzato et al. assessed endothelial function using serum markers in 10 children with acute leukemia and 11 children with non-lymphoblastic leukemia, before and after stem cell transplantation (SCT). In the aplastic phase after SCT, the endothelial selectin (ES) and leukocyte selectin (LS) dramatically lowered and reached pre-SCT values 4 weeks post-SCT. They showed that ES and LS were higher in Allo-SCT than Auto-SCT, reflecting the major white blood cell (WBC) count after Allo-SCT, which was in accordance with the close interrelation between leukocyte/inflammation and endothelial dysfunctions. The NO_2_/NO_3_ ratio significantly increased following SCT. Following SCT increases in thrombomodulin (TM) and vWF serum levels did not reach statistical significance, while EN and TF did not change significantly. Their observation supports previous data of severe endothelial damage after conditioning and SCT. The increase in nitric oxide (NO) metabolites, with protective action on the endothelium, may reflect the regeneration of the endothelium after a transitory functional impairment. Despite their central role in microvascular damage, coagulation proteins did not appear to be useful markers of endothelial function in SCT. Important limitations of the study include a small population and no control group, as well as the fact that no sample baseline metabolic characteristics are available [78].

A subsequent study [80] in larger populations confirmed a significant increase in TM and vWF during the acute phase of ALL and the remission of the disease. In the same study, patients in the acute phase had elevated acute phase protein levels as compared with both (ALL complete remission and healthy) control groups, and decreased levels of β2-integrins as compared with the HC group. Patients in complete remission had increased levels of soluble P-selectin as compared with the acute phase and HC groups, and decreased levels of β2-integrins as compared with both control groups. The increased levels of soluble P-selectin in children during treatment and in the ALL-control group suggest the presence of endothelial dysfunction that possibly results from oncological treatments. Patients who relapsed or died had higher leukocyte counts and TM levels at diagnosis as compared with patients in sustained remission. The significantly elevated levels of vWF and TM in patients with ALL during the acute phase and remission of the disease confirm the presence of endothelial dysfunction in ALL. Moreover, endothelial dysfunction in children with ALL at the time of diagnosis is likely caused by the disease itself, while endothelial dysfunction during remission probably results from oncological treatment. Furthermore, the positive correlation between leukocyte count and levels of both TM and vWF before treatment as well as the high levels of TM in children with an unfavorable outcome suggest that TM and vWF levels might represent additional prognostic markers of childhood ALL, but long-term follow-up is needed.

A recent case-control study was conducted in 100 childhood ALL survivors and 80 healthy age- and sex-matched children as a control group, to assess the endothelial dysfunction in ALL survivors using a new serum endothelial-specific molecule, i.e., endocan. ALL survivors showed statistically higher serum endocan levels and this was positively correlated with the classic parameters of endothelial dysfunction such as cIMT and lipid profile [93].

In order to investigate the influence of 25-hydroxyvitamin D (25-OHD) levels on vascular function in ALL survivors, Muggeo et al. [89] evaluated the biochemical and hemostatic markers of endothelial function (ET-1, high-molecular weight adiponectin, thrombin–antithrombin complex, vWF antigen, D-dimers, fibrinogen, and high-sensitive C-reactive protein) and ultrasound markers of vascular endothelial function (FMD, cIMT, and the anteroposterior diameter of the infrarenal abdominal aorta); 52 ALL survivors and 40 matched HCs were compared. They found a higher prevalence of 25-OHD deficiency (<20 ng/m) in CCS and a significant negative association between 25-OHD levels and vascular function in CCS evaluated as cIMT. In CCS, the higher 25-OHD levels seemed to be associated with a reduction in cIMT and higher levels of ET-1 and high-molecular weight adiponectin (HMW-AD). In multivariable additive regressions, adjusting for the confounding effect of both BMI and LDL-C, the 25-OHD levels in CCS were still negatively associated with cIMT and ET-1, and positively with HMW-AD. Although limited by the small sample size, the conflicting result between vitamin D and cIMT/ET-1 might be an expression of endothelial homeostasis (in the cells experimental study, vitamin D increases the levels of ET-1 and NO [100]). This finding confirms that the relationship between endothelial dysfunction and endothelial biomarkers is complex and not yet entirely clear.

## 3. Discussion

For decades, vascular endothelium was thought to be a single layer of cells without an active role in the transfer of water or other molecules. Today, it is known that the endothelium is a dynamic barrier that regulates the transfer of small and large molecules through active interaction with circulating cells and soluble blood molecules.

**Figure 2:**
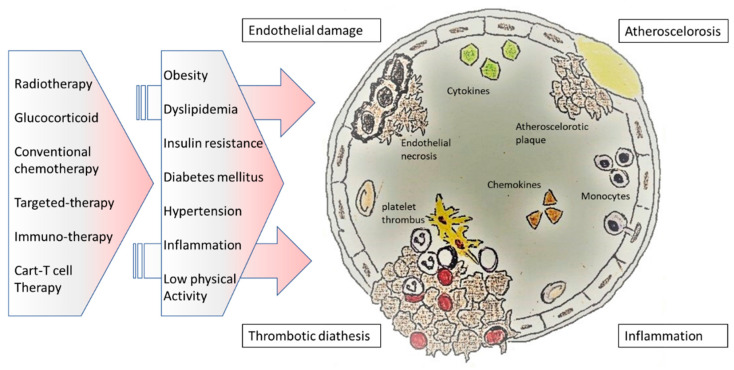
Etiopathogenesis of endothelial dysfunction in cancer survivors.

Endothelial damage may be the result of an overlap of mechanisms directly related to the early and late effects of cancer and/or oncological therapies (Figure 2). Indeed, a decline in endothelial function of patients after cancer can occur as a consequence of direct cytotoxic effects of tumors or cancer therapies on the endothelial cells. Secondary damage may be the result of a reduction in microvascular and endothelial functions, due to reactive inflammatory vasculitis, remodeling of microvascular architecture, and chronic damage linked to cardiometabolic risk factors [6].

Cancer therapy has evolved remarkably, from chemical compounds in the twentieth century to targeted agents and immunotherapies over the last two decades. With these developments, the new cardiovascular toxicity profiles of cancer therapeutics are broadening, and therefore becoming the subject of new studies.

Numerous conventional chemotherapies have been associated with adverse effects and complications across the whole cardiovascular system. Radiotherapy may amplify the chemotherapy damage, impair the endothelial cells, and cause arterial stiffness through alterations in microvascular structure [101]. Despite a sudden increase in the availability of new target therapies, there is little data on endothelial adverse events and a lack of studies on the direct and indirect mechanisms implicated in vascular toxicity due to these novel targeted agents [102]. Recent evidence has shown the involvement of target therapies in cardiovascular risk. New target therapies may be the cause of vascular erosion through the loss of the endothelial monolayer involved in apoptosis or necrosis. This endothelial damage is often coupled with impairment of repair mechanisms. The administration of vascular endothelial growth factor (VEGF) inhibitors and multi-target tyrosine kinase inhibitors can inhibit proliferation and migration of neighboring endothelial cells [103]. Inflammation may be stimulated by increased cytokine levels, for example, as a result of immune checkpoint inhibitor therapy on leukocytic cells or an increase in proinflammatory cytokines with chimeric antigen receptor T-cell [6,104].

Moreover, endothelial dysfunction may be the consequence of endocrinopathy (the most common growth hormone deficiency) and an imbalance in the physiological feedback between the mechanical stimuli and vasoactive agents (NO and prostacyclin) which aim to regulate the homeostasis of the vasomotor tone, endothelial permeability, vascular flow, and blood cells adhesion. Endothelial and vascular long-term toxicity may also occur two decades after the end of cancer therapy, especially due to the high risk of late effects such as visceral adiposity and MS. The metabolic derangements, featured by a proinflammatory state, may negatively affect endothelial cell function and worsen the microvascular damage related to dyslipidemia, insulin resistance, and hypertension [105] (Figure 2). Assessment of endothelial function may identify asymptomatic subjects at high risk of cardiovascular events and who could benefit from multidisciplinary management and early intervention, if necessary, with cardioprotective drugs.

Recently, many non-invasive alternative approaches for assessing endothelial function have been evaluated. Most of these methods have been developed to explore systemic endothelial function through ultrasonographic markers.

The use of cIMT measurements in routine clinical practice is limited by the need for personnel training, the availability of appropriate ultrasonographic equipment, and the lack of uniformly accepted measurement protocols, which generate difficulties in comparing the results of various studies. Additionally, although there are multiple studies on PWV in large populations of healthy children and adolescences, the studies all suffer from notable limitations, primarily, the lack of ethnic- and pubertal stage-dependent normative data, the heterogeneity of measurement devices, and protocols of scanning.

FMD is easy to use and has shown good reliability in adults; therefore, ultrasound methods such as FMD are often used in child patients. Nevertheless, FMD is strongly operator dependent, and results can be invalidated by observer variability. Moreover, reference ranges were proposed by several authors, but with some discrepancies between the different publications. Until better pediatric reference ranges will be available, a control group is needed in the childhood age studies. Other lesser-used methods include the evaluation of vascular wall and microvascular structures, arterial stiffness, finger arterial pulsatile volume changes or circulating blood markers. In the absence of reliable pediatric normative data, these methods remain as research tools in the pediatric population.

## 4. Conclusions

The need for screening, treatment, and prevention of vascular toxic effects of anticancer therapies is now supported by consolidated data. Increased awareness of the vascular toxic effects of chemotherapy and radiotherapy has further revealed the urgent need to define the best clinical practices to complement the classic cardiovascular risk markers (glycolipid profile and anthropometric measures).

Over the last two decades, several methods have been described and have been used to assess the functional state of endothelium. However, the gold standard method continues to be the endothelial vasomotor testing performed with intracoronary administration of vasoactive reagents such as acetylcholine. Nevertheless, considering that cardiovascular impairment may appear even more than 20 years after the end of oncological treatment, the invasive test raises serious ethical concerns regarding its application on a broader scale, especially in children.

Due to numerous studies published about children and the availability of reliable pediatric reference ranges supported by scientific associations, currently, the most recommended methods are cIMT and PWV measurements. However, pediatric reference ranges and uniformly accepted measurement protocols are still lacking.

Future studies are needed to provide additional insight into the pathophysiology of vascular disease and the vascular nature of cardiotoxicity of new anticancer therapies such as targeted biological therapies, and new radiotherapy techniques such as proton therapy.

## Figures and Tables

**Table 1:** Endothelial dysfunction results: Cancer characteristics of the studies.

Study Descriptions and Cancer Characteristics						
First Author, Year, [Reference]	Aim of Study	Population, Age Mean ± SD or Median (Interquartile Range) N (F/M)	Cancer Characteristics and Treatments	Endothelial Dysfunction Assessment	Endothelial Dysfunction Results Mean ± SD or Median (Interquartile Range) * *p* < 0.05 ** *p* < 0.005 ^ Not-significant (+): r > 0	Outcomes
Luzzato, 2003 [78]	Evaluation of serum endothelial markers before and after SCT in children with acute leukemia	**CS:** 7.5 ± 5.1 years N = 21 (F/M = 9/12)	**SCT for acute leukemia** = 21 (10 ALL, 11 non-lymphoblastic) **SCT divided in:** **8/21 autologous** (7 from bone marrow and 1 from peripheral blood stem cells) **13/21 allogenic** (3 from related donor and 10 from unrelated donor) **Pre-SCT conditioning:** 8/21 TBI + TT + CYC + ATG 5/21 TBI + MPH 2/21 TBI + TT + CYC 2/21 BU + CYC+ ATG 2/21 TBI + ARA-C 1/21 M 1/21 BU + CYC **After SCT:** **10/21** no complications **11/21** major complications (2 death, 5 severe infections, 4 VOD)	**Circulating blood markers** ES, LS, TM, vWF, NO_2_/NO_3_, ET-1, TF before SCT, immediately after SCT and four weeks after SCT	**ES and LS:** pre-SCT > immediately post-SCT ** NO_2_/NO_3_: pre-SCT < immediately post-SCT **, and four week post-SCT ** TM and **vWF:** pre-SCT < post-SCT ^ **ET-1 and TN:** pre-SCT = immediately post-SCT and four week post-SCT **WBC:** Allo-SCT > Auto-SCT * ES after 4 weeks SCT (ng/mL): allo-SCT 66.1 ± 15.7 vs. auto-SCT 22.2 ± 2.8 * LS after 4 weeks SCT (ng/mL): allo-SCT 558.8 ± 89 vs. SCT 241 ± 11.1 * WBC correlate (+) with ES and LS ** TM correlate (+) with ES, LS and NO_2_/NO_3_ **	Conditioning and SCT cause severe endothelial damage ES and LS lower immediately after SCT. ES and LS returned to pre-SCT levels after 4 weeks post SCT ES and LS higher in Allo-SCT than Auto-SCT reflecting the major WBC counts after Allo-SCT Increase in TM and NO metabolites may reflect endothelial regeneration after SCT TF not a useful marker of endothelial damage
Chow, 2006 [79]	Assessment of endothelial toxicity caused by AAs in CCS	**CS:** 14.5 ± 4.45 years N = 14 (F/M = 5/9) **HC:** 11.1 ± 5.11 years N = 14 (F/M = 5/9) **Months since off therapy:** 19.8 ± 18.7	**T-Cell ALL** = 3 **AML** = 3 **APML** = 1 **Lymphoma** = 1 **Ewing Sarcoma** = 3 **Osteosarcoma** = 1 **Abdominal sarcoma** = 1 **All treated with AAs cumulative dose** > 300 mg/m^2^; **RT** = 7 (1 TBI, 2 pelvis, 3 brain, 1 chest)	**FMD** at rest and 1′ after blood pressure cuff occlusion	**FMD (%)**: CS 3.8 ± 3.4 vs. HC 6.7 ± 3.3 *	FMD is lower in cancer survivors AAs cause impaired endothelial function associated with progression in coronary disease
Hatzipantelis, 2011 [80]	Evaluation of markers of endothelial activation in children with ALL and assessment of their prognostic value	**ALL acute phase:** 6.4 (1–13) years N = 52 (F/M = 19/33) **ALL complete remission** (33rd day since off-therapy) N = 49 (3 pts died during acute phase) (F/M N.A.) **ALL relapsed or died** N = 13 (F/M N.A.) **ALL sustained remission** N = 39 **ALL control group** (full remission, 1–10 years since off-therapy): 14.1 (6–18) years N = 19 (F/M N.A.) **HC:** 6.4 (2.5–14) years N = 28 (F/M N.A.)	**B-cellALL Acute Phase** = 45 **T-cell** **ALL Acute Phase** = 7 **Treatment:** **UCALL-XI protocol** = 12 **BFM-95 protocol** = 40	**Acute phase reactants** ESR, CRP, IL-6 **Endothelial factors** NO, ET-1, PDGF, vWF, TM **Adhesion molecules** P-selectin, VCAM-1, B2-integrins, LFA1-2-3	**ESR (mm/h):** ALL-AP 67.1 ± 6.3 vs. ALL-CG 6.3 ± 1.1 ** o HC 5.8 ± 0.7 ** CRP (mg/dl): ALL-AP 10.2 ± 2.9 vs. ALL-CG 0.3 ** o HC 0.2 ** IL-6 (pg/mL): ALL-AP 11.6 ± 2.4 vs. ALL-CG 4.2 ± 0.7 * o HC 4.3 ± 0.7 * TM (ng/mL): ALL-AP 23.2 ± 3.4 vs. ALL-CG 10.9 ± 2.9 * o HC 10 ± 3.6 * TM (ng/mL): ALL CR 20.2 ± 3.4 vs. ALL-CG 10.9 ± 2.9 * o HC 10 ± 3.6 * vWF (%): ALL-AP 164 ± 612 vs. ALL-CG 103.9 ± 10.5 ** o HC ± 99.7 9.1 ** vWF (%): ALL CR 174.3 ± 15 vs. ALL-CG 103.9 ± 10.5 o HC 99.7 ± 9.1 ** TM (ng/mL): ALL Relapsed or died 30 ± 8.6 vs. sustained remission 20.8 ± 3.4 * TM (ng/mL) and vVF (%): ALL-CR vs. relapsed/died ^ o sustained remission ^ **P-Selectin (pg/mL):** ALL-CR 172.6 ± 28 vs. ALL-AP 80.4 ± 11.5 * o HC 79.6 ± 6.9 * P-Selectin (pg/mL): ALL-CG 176.1 ± 9.4 vs. ALL-AP 80.4 ± 11.5 * o HC 79.6 ± 6.9 * LFA-1 (%): ALL-AP 56.6 ± 3.5 vs. HC 79.6 ± 6.9 ** LFA-1 (%): ALL-CR 67.3 ± 3.0 vs. ALL-CG 71.9 ± 4.5 ** o HC 79.6 ± 6.9 ** LFA-2 (%): ALL-AP 75.7 ± 2.8 vs. HC 79.6 ± 6.9 ** LFA-2 (%): ALL-CR 85.4 ± 2.0 vs. ALL-CG 88.6 ± 2.4 * o HC 92.5 ± 1.0 * LFA-3 (%): ALL-AP 50.0 ± 4.1 vs. HC 80.4 ± 11.5 ** LFA-3 (%): ALL-CG 63.0 ± 4.1 vs. HC 80.4 ± 11.5 *	High levels of vWF and TM in acute phase and remission confirm endothelial dysfunction in ALL Patients died/relapsed had higher TM at diagnosis than patients with sustained remission TM and vWF might represent additional but not independent prognostic markers of ALL Increased P-selectin suggest that endothelial dysfunction may results from chemotherapy
Herceg-Cavrak, 2011 [81]	Evaluation of arterial stiffness after treatment with AAs	**CS:** 13.6 ± 4.4 years N = 53 (F/M = 19/34) Time since off therapy: 2 (1–16) years **HC:** 12.2 ± 3 years N = 45 (F/M = 20/25)	**Ewing sarcoma** = 17 **NHL + HGD** = 9 **Wilms tumor** = 4 **Neuroblastoma** = 4 **Synovial sarcoma** = 3 **Rhabdomyosarcoma** = 3 **Not specified tumors** = 13 **Treatment:** **AAs** CD 212 ± 93 mg/m^2^ **CYC** CD 4.4 ± 3 g/m^2^ **Others CHT:** MTX, Alkaloid Vincristine, Cisplatin	**PWVao** **Arterial stiffness markers** PPao, SBPao, MAP	**PWVao (m/s):** CS 6.24 ± 1.34 vs. HC 5.42 ± 0.69 ** PWVao (m/s): CS treated with CYC 6.41 ± 1.34 vs. HC 6.21 ± 1.17 ^ **PWVao (m/s):** CS females 6.1 ± 1.34 vs. CS males 6.33 ± 1.35 ^ **PWVao (m/s):** HC females 5.5 ± 0.6 vs. HC males 5.35 ± 0.8 ^ **PPao (mmHg), SBPao (mmHg), MAP (mmHg):** CS vs. HC ^	PWVao significantly increased in patients treated with AAs Cardiovascular morbidity in CS treated with AAs could be related to vascular stiffness, not only to cardiotoxicity No correlation between PWVao with the dose of AAs
Jang, 2013 [82]	Evaluation of endothelial function in Korean children affected by ALL treated with AAs	**CS:** 10.3 ± 4.3 years N = 21 (F/M = 10/11) Time since off therapy: 2–85 mm **HC:** 9.6 ± 4.1 years N = 20 (F/M = 11/9)	**ALL** treated with AAs 142.5 ± 18.2 mg/m^2^ **Other CHT:** Vincristine, Prednisolone, MTX, 6-Mercaptopurine, L-asparaginase, Cyclophosphamide	**FMD** at rest and 1′ after blood pressure cuff occlusion	**FMD (%):** CS 3.4 ± 3.9 vs. HC 12.1 ± 8.0 *	AAs cause endothelial function impairment in ALL children and play an important role in the progression of CVD No correlation between BAR and elapsed time after the last AAs administration and age at AAs administration
Jenei, 2013 [83]	Evaluation of endothelial-dependent and independent vascular function and arterial stiffness simultaneously in individuals who received CHT containing AAs, different chemotherapy and comparison with HC.	**CS:** 14.9 ± 5.3 years N = 96 (F/M = 39/57) Time since off therapy at least 5 years CS divided according to CT: **CS CHT + AAs group:** 15.1 ± 4.2 years N = 67 (F/M = 28/39) Time since off therapy: 11.2 ± 6.3 years; **CS CHT group:** 14.7 ± 5.1 years N = 29 (F/M = 11/18) Time since off therapy: 10.8 ± 5 years; **HC:** 13.7 ± 4.9 years N = 72 (F/M = 33/39)	**ALL** = 49 **AML** = 2 **HGD/NHL** = 12 **Treatment BFO protocol:** AAs, HD MTX, CYC and IFO. **CRT < 24 Gy** = 21 **WT** = 14 **Treatment**: 12 Vincristine and D-Actinomycine, 2 received also IFO and CB, 1 RT **ST** = 19 (11 Neuroblastoma, 5 Osteosarcoma, 2 Ewing sarcoma, 1 Schwannoma) **Treatment:** 14 AAs, IFO or PD or HD MTX	**FMD** **Arterial stiffness markers** NTG, SI-B, aortic distensibiliy.	**FMD (%):** AAs 7.1 ± 6.3 vs. CHT 10.2 ± 4.2 * o HC 13.1 ± 2.4 * FMD (%): AAs 10.2 ± 4.2 vs. HC 13.1 ± 2.4 * FMD (%): AAs 7.1 ± 6.3 vs. CHT 10.2 ± 4.2 * FMD (%): CHT 10.2 ± 4.2 vs. HC 13.1 ± 2.4 * FMD peak (%): female AAs 8.1 vs. male AAs 6.1 * FMD peak (%): female CHT 11.2 vs. male CHT 9.1 * FMD peak (%): female HC 14.1 vs. male HC 12.1 * NTG (%): HC 26.3 ± 6.1 vs. AAs 25.9 ± 4.4 ^ o CHT 25.9 ± 5.7 ^ **SI-B**: AAs 6.4 ± 3.2 vs. CHT 4.1 ± 2.3 * o HC 2.1 ± 0.6 * SI-B: AAs 6.4 ± 3.2 vs. HC 2.1 ± 0.6 * SI-B: AAs 6.4 ± 3.2 vs. CHT 4.1 ± 2.3 * SI-B: CHT 4.1 ± 2.3 vs. HC 2.1 ± 0.6 * FMD correlate (−) with TG levels *, age *, AA CD **, aortic distensibility ** FMD correlate (+) with age of starting treatment * Aortic distensibility correlate (−) with TG levels *, AA CD ** Distensibility correlate (+) with age of starting treatment * SI-B correlate (−) with age of starting treatment * SI-B correlate (+) with TG levels ** and AAs CD **.	First study demonstrating a link between endothelial dysfunction and aortic stiffness in CS Long-term CS exposed to AAs treatment with mean CD 242 ± 56 mg/m^2^ exhibit preclinical vasculopaty, endothelial dysfunction and vascular stiffness Endothelial disfunction persistsfor more than 10 years after AAs treatment AAs CD doses, age at treatment and TG levels add negative effects on endothelial function and stiffness FMD% significantly lower in both CS groups than in HC FMD% significantly lower in AAs vs. CT without AAs Peak of FMD% higher in females than in males among 3 groups No gender differences in other parameters NTG% not significantly different among 3 groups SI-Beta worst in CS than HC and in AAs than CHT group
Blair, 2014 [84]	Evaluation of flavanoid-rich purple grape juice (RCCT with clear apple juice) on microvascular endothelial function and markers of oxidative stress and inflammation in CS	**CS**: 16.4 (13.7–17.2) years N = 24 (F/M = 17/7) Age at cancer diagnosis: 3.6 (1.5–6.1) years Time since off therapy: 8.5 (6.4–13) years	**Solid tumor** = 12 (3 CNS, 3 bone, 2 retinoblastoma, 2 GCT, 1 neuroblatoma, 1 hepatoblastoma, 1 soft tissue sarcoma) **Hematopoietic malignancy** = 12 (9 ALL, 1 AML, 1 HGD, 1 NHL) **CT + RT** = 5 **CT** = 14 (16 alkylating agents, 15 AAs, 11 antimetabolites, 5 platinum agents, 5 topoisomerase inhibitors, 2 antibiotics) **RT** = 2 (5 RT head, 2 spine, 1 chest, 1 TBI) **Surgery only** = 3	**RH-PAT** **Circulating blood markers** OxLDL, MPO, hs-CRP	**RH-PAT:** before apple juice 1.57 ± 0.36) vs. before grape juice 1.75 ± 0.52 **RH-PAT:** after apple juice 1.83 ± 0.47 vs. after grape juice 1.75 ± 0.39 ^ **RH-PAT:** before grape juice 1.57 ± 0.52 vs. after grape juice 1.75 ± 0.39 ^ **Ox LDL (U/L):** before apple juice 66.2 ± 13.4 vs. before grape juice 61.7 ± 15.1 ^ **Ox LDL (U/L):** after apple juice 66.6 ± 13.8 vs. after grape juice 66.7 ± 17.2 ^ **Ox LDL (U/L):** before grape juice 61.7 ± 15.1 vs. after grape juice 66.7 ± 17.2 ^ **MPO (ng/mL):** before apple juice 116.2 (93–142) vs. before grape juice 117.3 (98–138) ^ **MPO (ng/mL):** after apple juice 116.2 (93–142) vs. after grape juice 107 (92–131) ^ **MPO (ng/mL):** before grape juice 117.3 (98–138) vs. after grape juice 107 (92–131) ^ **hs-CRP (mg/L):** before apple juice 0.24 (0.07–0.55) vs. before grape juice 0.19 (0.09–0.41) ^ **hs-CRP (mg/L):** after apple juice 0.24 (0.11–0.85) vs. after grape juice 0.33(0.15–0.73) ^ **hs-CRP (mg/L):** before grape juice 0.19 (0.09–0.41) vs. after grape juice 0.33 (0.15–0.73) ^	After four weeks of daily consumption of flavanoid-rich purple grape juice, no significant change in vascular function was observed in young, relatively healthy CS.
Dengel, 2014 [85]	Measurement of carotid and brachial artery structures and function in CS during childhood	**CS:** 14.6 ± 0.1 years N = 319 (F/M = 96/112) Time since off therapy: 10.1 ± 0.2 years White non-Hispanic/Others: 194/28 **CS divided in 3 groups** according to kind of tumor **HC:** 13.6 ± 0.2 years N = 208 (F/M = 148/171) White non-Hispanic/Others: 274/90	**Leukemia** = 110 (102 ALL, 8 AML) **Treatment:** CRT = 14 **CNS** = 82 (38 Glial tumors, 16 Retinoblastoma, 13 Neuroectodermal tumors, 15 other) **Treatment:** CRT = 26 **ST** = 127 (32 Sarcomas, 30 Renal tumors, 23 Neuroblastom, 20 NHGL, 22 others) **Treatment:** CRT = 4 Not brain RT = 26	**cIMT** **Arterial stiffness markers** Brachial artery EDD, peak NTG-mediated EID, DD and CSD	**EDD (%):** CS 7.6 ± 0.3 vs. HC 8.2 ± 0.4 ^ **EDD (%):** Leukemia 7.5 ± 0.4 vs. HC 8.2 ± 0.4 * EID (%): CS 25.1 ± 0.6 vs. HC 26.2 ± 0.4 ^ **C-IMT (mm):** CS 0.44 vs. HC 0.44 ^ **C-IMT (mm):** CNS 0.45 vs. Leukemia 0.44 * C-IMT (mm): Leukemia 0.44 vs. ST 0.44 and vs. HC 0.44 ^ **DD and CSD:** CS < controls ** DD and CSD: CNS < controls * DD, CSD, DC, CSC, IEM: CS vs. controls ^	Early in life CS have arterial changes indicating increased risk of premature atherosclerosis and CVD Significantly lower measure of vascular function in carotid and brachial arteries in Leukemia survivors indicating arterial stiffness EDD similar in HC and all CS, but is significantly lower in group of leukemia than HC EID similar in CS and HC Carotid-IMT ticker in CNS survivor than control and leukemia, but not different between all CS and HC and between leukemia and Solid tumor group
Krystal, 2015 [86]	Evaluation of PWV in a cohort of CS and HC	**CS:** 17.3 ± 6 years N = 68 (F/M = 32/36) Age at cancer diagnosis: 8.8 ± 5.5 years Time since off therapy: 7 ± 4.2 years **CS > 18 years** N = 30 **HC:** 18.5 ± 5.5 years N = 51 (F/M = 32/19) **HC > 18 years** N = 27	**ST** = 15 **Neuroblastoma** = 4 **Ewing sarcoma** = 3 **Osteosarcoma** = 1 **Hepatoblastoma** = 1 **Wilms tumor** = 6 **Hematopoietic malignancies** = **53** (17 HGD, 6 NHL, 28 ALL, 2 AML **Treatment:** **CT** = 68 (54 alkylating agents, 68 AAs, 1 platinum agents, 28 topoisomerase inhibitors, 15 antibiotics, 49 steroids) **RT** = 34 (4 TBI, 12 abdomen/pelvis, 13 head/neck, 2 extremity, 3 others: MIBG, testicular, sacral)	**PWV**	**PWV:** CS 5.74 ± 1.10 vs. HC 5.65 ± 88 ^ **PWV:** CS > 18 years 6.37 ± 0.89 vs. HC > 18 years 5.76 ± 0.88 * PWV: CS < 18 years 5.23 ± 0.99 vs. HC < 18 years 5.5 ± 0.87 ^	CS and HC had similar PWV overall Subgroup analysis revealed that CCS older than 18 had significantly higher PWV than HC older than 18, also when analyzed for age, gender, and BMI z-score Only exposure to radiation therapy and time off therapy were significantly associated with greater PWV
Okur, 2016 [87]	Detection of subclinical atherosclerosis and endothelial function in children with solid tumor treated with AAs	**CS:** 13.5 ± 4.7 years N = 50 (F/M = 15/35) **HC:** 12.0 ± 4.3 years N = 30 (F/M = 12/18)	**HGD** = 27 **NHL** = 6 **Solid Tumors** = 17 (3 osteosarcoma, 4 Ewing sarcoma, 5 Wilms tumor, 1 Hepatoblastoma, 4 Neuroblastoma) **Treatment:** **AAs** = 50 divided in groups of CD Group 1 = 19: <100 mg/m^2^ Group 2 = 19: 101–299 mg/m^2^ Group 3 = 12: >299 mg/m^2^ **RT** = 36 **RT** 25.3 (10.8–54) Gy	**FMD** **cIMT** **Adhesion molecules** sICAM, sVCAM, ES	**FMD (%):** CS 7.4 ± 9.3 vs. HC 8.3 ± 4.6 ^ **FMD (%):** AAs CD >300 mg/m^2^ 3.1 ± 5.9 vs. HC 8.3 ± 4.6 ** FMD (%): AAs CD < 100 mg/m^2^ 10.4 ± 9.9 vs. AAs CD > 300 mg/m^2^ 3.1 ± 5.9 ** C-IMT (mm): CS 0.51 ± 0.1 vs. HC 0.47 ± 0.1 * sICAM (ng/mL): CS 432 ± 100 vs. HC 419 ± 100 ^ **sVCAM (ng/mL):** CS 1510 ± 792 vs. HC 1575 ± 618^ **ES (ng/mL):** CS 57.2 ± 31 vs. HC 55.1 ± 33 ^	AAs lead endothelial dysfunction as the cumulative dose increases BAR similar between HC and CS BAR worsened if CD of AAs increased Carotid IMT higher in CS vs. HC IMT non influenced by AAs CD and RT No difference for sICAM, sVCAM and E selectine between CS, HC and cumulative dose of AAs
Masopustová, 2018 [88]	To determine whether a significant difference in RHI is found in pediatric ALL survivors as compared to controls; To discern if the association between RHI and specific biochemical markers in ALL survivors exists; and to demonstrate whether the combination of RHI and biochemical parameters can be used for the detection of ED in pediatric ALL survivors.	**ALL survivors:** 15.6 (12.72–17.95) years N = 22 (F/M = 7/15) Time since off therapy: at least two years **HC:** 16.1 (12.91–17.33) years N = 18 (F/M = 13/5)	**Treatment** BFM ALL 95 or ALL IC-BFM 2002 protocols AAs CD 240–360 mg/m^2^ CYC CD 3000 mg/m^2^	**RH-PAT** **Circulating blood markers** ADMA, ES, VCAM, hs-CRP	**RH-PAT:** CS 1.5 (1.3–2.0) vs. HC 1.8 (1.59; 2.46) *. **hs-CRP (mg/L):** CS 1.1 (0.71–2.29) vs. HC 0.19 (0.18–0.45) ** ES (μg/L): CS 76.0 (58.32–108.98) vs. 62.5 (31.66; 70.99) * ADMA (µmol/L): CS 0.6 (0.53–0.66) vs. HC 0.58 (0.49–0.61) ^ **VCAM (µg/L):** CS 941.7 (818.66–1074.0) vs. HC 918.4 (793.08–1017.90) ^	Significantly decreased RH-PAT, elevated plasma levels of hs-CRP and E-selectin support hypothesis of increased risk of premature ED in these patients. The combined approach seems to be a promising method for the assessment of endothelial function.
Muggeo, 2019 [89]	Investigate the 25-OHD status in children treated for ALL, and its influence on vascular function. 25-OHD deficiency considered if levels were <20 ng/mL.	**ALL survivors:** 9.7 ± 4.1 years N = 52 (F/M = 33/19) Time since off therapy: 28.2 (4–102) mm **HC:** 10.5 ± 4 years N = 40 (F/M = 24/16)	**Treatment** AIEOP-BFM protocol Standard risk = 18 Medium risk = 29 High risk = 5	**FMD** **cIMT** **APAO** **Circulating blood markers** HMW-AD, ET-1, vWFA, TAT, D-dimers, Fbg, hs-CRP	**FMD** (%): CS 25-OHD < 20 ng/mL 10.5 ± 4.8 vs. CS 25-OHD > 20 ng/mL 8.8 ± 3.8 ^ **cIMT (mm):** CS 25-OHD < 20 ng/mL 0.5 ± 0.1 vs. CS 25-OHD > 20 ng/mL 0.4 ± 0.1 * APAO (cm): CS 25-OHD < 20 ng/mL 10.0 ± 2.2 vs. CS 25-OHD > 20 ng/mL 10.1 ± 1.8 ^ **HMW-AD (μg/mL):** CS 25-OHD < 20 ng/mL 5.1 ± 2.5 vs. CS 25-OHD > 20 ng/mL 3.4 ± 2.0 * ET-1 (pg/mL): CS 25-OHD < 20 ng/mL 2.0 ± 0.6 vs. CS 25-OHD > 20 ng/mL 2.3 ± 0.6 * TAT (μg/L): CS 25-OHD < 20 ng/mL 3.9 ± 4.8 vs. CS 25-OHD > 20 ng/mL 3.7 ± 3.8 ^ **vWFA (%):** CS 25-OHD < 20 ng/mL 90.7 ± 19.5 vs. CS 25-OHD > 20 ng/mL 89.0 ± 16.7 ^ **D-dimers (ng/dL):** CS 25-OHD < 20 ng/mL 297.6 ± 152.4 vs. CS 25-OHD > 20 ng/mL 363.9 ± 204.9 ^ **Fbg (mg/dL):** CS 25-OHD < 20 ng/mL 265.6 ± 48.1 vs. CS 25-OHD > 20 ng/mL 261.4 ± 36.8 ^ **hs-CRP (mg/L):** CS 25-OHD < 20 ng/mL 6.2 ± 13.0 vs. CS 25-OHD > 20 ng/mL 3.7 ± 1.4 ^	Childhood ALL survivors show higher prevalence of 25-OHD deficiency as compared with HC (62.2 % vs. 15 %) ** In LLA survivors 25-OHD levels linked to some indicators of endothelial and vascular dysfunction (HMW-AD, ET-1 and cIMT). Careful monitoring of 25-OHD balance may help to prevent cardiovascular diseases in childhood ALL survivors, characterized by high cardiovascular risk
Von Korn, 2019 [90]	Assessment of functional limitations in HRPF and cardiovascular risk by means of markers of arterial stiffness in CS (as compared with healthy reference peers)	**CS:** 12.5 ± 4.2 years N = 92 (F/M = 43/49) Age at cancer diagnosis: 8.8 ± 4.8 years Time from diagnosis: 3.6 ± 2.8 years	**Leukemia** = 54 **Solid tumors** = 28 **Lymphomas** = 10 **Treatment:** **CT** = 89 (73 AAs, dose 225 ± 83 mg/m2) **RT** = 10 (dose 28.4 ± 19.9 GY) **RT + CT** = 4 (AAs CD 248.0 ± 89.9 mg/m2; radiation dose 18.4 ± 8.5 GY **Surgery or only wait and see** = 3	**PWV** **HRPF** **Arterial stiffness markers** PSBP, PDBP, CSBP	**PWV z-score:** 0.1 ± 1.4 ^ **HRPF z-score:** −0.3 ± 1.0 * PSBP z-score: 0.3 ± 1.1 * PDBP z-score: −0.3 ± 1.2 * CSBP z-score: 0.1 ± 1.3 ^ Comparison of PWV and CSBP to the German reference from the Elmenhorst et al. [60] Comparison of PSBP, PDBP to the German reference from the German KIGGS Study [91]	Increased pulse pressure as a result of increased PSBP and decreased PDBP in CCS These findings may reflect subtle early changes of arterial wall stiffness, which not yet detected by arterial stiffness parameters PWV and CSBP, still within the expected range No significant difference was showed between patients treated AAs and patients who did not receive cardiotoxic therapy
Keiser, 2020 [92]	Investigating specific parameters as early predictors of potential damage to the cardiovascular system after cancer treatment	**CS:** 11.28 ± 3.8 years N = 40 (F/M = 20/20) Divided in 2 groups: <8 years = 10 ≥8 years = 30 Age at cancer diagnosis: 8.26 ± 4.32 years Time since off therapy: 1.56 ± 1.79 years <1 year = 19 (48%) 1–5 years = 20 (50%) >5 years = 1 (3%)	**Leukemia/Lymphoma** = 18 **Bone tumor** = 2 **Brain tumor** = 7 **Alveolar rhabdomyosarcoma** = 1 **Carcinoid tumor of the appendix** = 2 **Nephroblastoma** = 3 **Liver focal nodular hyperplasia** = 1 **Ovarian mature cystic** **teratoma** = 2 **Thoracic ganglioneuroma** = 2 **Thyroid papillary carcinoma** = 1 **Neuroblastoma** = 2 **Treatment:** **CT** = 27 (AAs = 25, CD 27 ± 81 mg/m^2^) **RT** = 13 (4 Chest-directed radiation, 4 AAs + chest radiation) **Surgery** = 19	**PWV** **Arterial stiffness markers** PSBP, PDBP, CSBP	**PWV:** <8 years z-score: 1.15 ± 2.89 ^ ≥8 years z-score: 0.55 ± 1.90 ^ **PSBP z-score:** 0.87 ± 1.67 ** PDBP z-score: 0.83 ± 1.94 * CSBP values: <8 years z-score: N.A.^ ≥8 years z-score: 0.60 ± 1.29 * Comparison of PWV and CSBP to the German reference from the Elmenhorst et al. [60] Comparison of PSBP, PDBP to the German reference from the German KIGGS Study [91]	Impaired cardiovascular function in children and adolescents shortly after cessation of cancer treatment PSBP and CSBP values significantly increased compared to reference values of healthy children and adolescents PWV elevated, but not significantly No association between increased blood pressure or PWV and AAs
Sherief, 2021 [93]	To assess endothelial dysfunction in ALL survivors using serum endocan and measurement of cIMT	**CS:** 10.7 ± 2.9 years N = 100 (F/M = 54/46) **HC:** 9.7 ± 2.7 years N = 80 (F/M = 40/40) Time since off therapy: at least ≥2	**Treatment:** B-line ALL = 80 T-line ALL = 20 Doxorubicin CD 180.5 ± 79.8 mg/m^2^	**cIMT** **Circulating blood markers** Serum endocan **Arterial stiffness markers** PSBP PDBP	**cIMT (mm):** CS 0.65 ± 0.13 vs. HC 0.32 ± 0.09 * Serum endocan (ng/L): CS 470.41 ± 556.1 vs. HC 225.94 ± 185.2 * PSBP: CS vs. HC ^ **PDBP:** CS vs. HC ^ **cIMT** correlate (+) with total cholesterol, LDL-Cand triglyceride levels * Serum endocan correlate (+) with cIMT and PDBP, correlate (−) with HDL *	Childhood ALL survivors showed serum endocan high levels and increased cIMT Serum endocan related with cIMT Serum endocan predictor of endothelial dysfunction and premature atherosclerosis

**Table 2:** Endothelial dysfunction results: Metabolic characteristics of the studies.

Metabolic Characteristics of Study Populations				
First Author, Year, [Reference]	Weight Mean ± SD or Median (Interquartile Range) BMI Mean ± SD or Median (Interquartile Range)	Systolic Blood Pressure Mean ± SD or Median (Interquartile Range) Diastolic Blood Pressure Mean ± SD or Median (Interquartile Range)	Fasting Glucose Mean ± SD or Median (Interquartile Range) Fasting Insulin Mean ± SD or Median (Interquartile Range) HbA1c Mean ± SD	LDL-C Mean ± SD or Median (Interquartile Range) HDL-C Mean ± SD or Median (Interquartile Range) Total Cholesterol Mean ± SD or Median (Interquartile Range) Triglycerides Mean ± SD or Median (Interquartile Range)
Luzzato, 2003 [78]	N.A.	N.A.	N.A.	N.A.
Chow, 2006 [79]	**CS** **W** (kg) = 52.9 ± 23.4 **BMI** (kg/m^2^) = N.A. **HC** **W** (kg) = 44.4 ± 27.2 **BMI** (kg/m^2^) = N.A.	**CS** **SBP** (mmHg) = 95.5 ± 5.7 **DBP** (mmHg) = N.A. **HC** **SBP** (mmHg) = 103.1 ± 15.9 **DBP** (mmHg) = N.A.	N.A.	N.A.
Hatzipantelis, 2011 [80]	N.A.	N.A.	N.A.	N.A.
Herceg-Cavrak, 2011 [81]	**CS** **W** (kg) = N.A. **BMI** (kg/m^2^) = 20.2 ± 4.6 **HC** **W** (kg) = N.A. **BMI** (kg/m^2^) = 18.9 ± 3.5	**CS** **SBP** (mmHg) = 109.7 ± 16 **DBP** (mmHg) = 61 ± 8.2 **HC** **SBP** (mmHg) = 114.4 ± 11.3 **DBP** (mmHg) = 63 ± 6.2	N.A.	N.A.
Jang, 2013 [82]	**CS** **W** (kg) = 43.5 ± 22 **BMI** (kg/m^2^) = N.A. **HC** **W** (kg) = 33.3 ± 14 **BMI** (kg/m^2^) = N.A.	**CS** **SBP** (mmHg) = 111.1 ± 15.6 **DBP** (mmHg) = N.A. **HC** **SBP** (mmHg) = 108.4 ± 10.9 **DBP** (mmHg) = N.A.	N.A.	N.A.
Jenei 2013, [83]	**CS CHT + AAs group** **W** (kg) = N.A. **BMI** (kg/m^2^) = 21.8 ± 4.8 **CS CHT group** **W** (kg) = N.A. **BMI** (kg/m^2^) = 20.1 ± 4.5 **HC** **W** (kg) = N.A. **BMI** (kg/m^2^) = 20.4 ± 2.9	**CS CHT + AAs group** **SBP** (mmHg) = 121.3 ± 3.5 **DBP** (mmHg) = 81 ± 2.1 **CS CHT group** **SBP** (mmHg) = 122 ± 4.1 **DBP** (mmHg) = 82 ± 1.4 **HC** **SBP** (mmHg) = 120 ± 5 **DBP** (mmHg) = 80 ± 3.1	**CS CHT + AAs group** **FG** (mg/dL) = 81.6 ± 14 **FI** (mU/L) = N.A. **HbA1c** (%) = N.A. **CS CHT group** **FG** (mg/dL) = 81.6 ± 16 **FI** (mU/L) = N.A. **HbA1c** (%) = N.A. **HC** **FG** (mg/dL) = 85.3 ± 9 **FI** (mU/L) = N.A. **HbA1c** (%) = N.A.	**CS CHT + AAs group** **LDL-C** (mg/dL) = 88.9 ± 27 **HDL-C** (mg/dL) = 54.1 ± 23 **Total cholesterol** (mg/dL) = N.A. **Triglycerides** (mg/dL) = 109 ± 83 **CS CHT group** **LDL-C** (mg/dL) = 81.2 ± 11.6 **HDL-C** (mg/dL) = 58 ± 35 **Total cholesterol** (mg/dL) = N.A. **Triglycerides** (mg/dL) = 113 ± 6 HC **LDL-C** (mg/dL) = 87.4 ± 22.8 **HDL-C** (mg/dL) = 61.7 ± 11.6 **Total cholesterol** (mg/dL) = N.A. **Triglycerides** (mg/dL) = 70 ± 26.4
Blair, 2014 [84]	**CS** **W** (kg) = 60.7 (50.4–72.1) **BMI** percentiles = 70.5 (47.1–82.3)	**CS** **SBP** (mmHg) = 111 (103–118) **DBP** (mmHg) = 59 (57–63)	**CS** **FG** (mg/dL) = 78 (57–83) **FI** (mU/L) = 5 (3–8) **HbA1c** (%) = N.A.	**CS** **LDL-C** (mg/dL) = 93 (78–111) **HDL-C** (mg/dL) = 50 (42–56) **Total cholesterol** (mg/dL) = 159 (142–177) **Triglycerides** (mg/dL) = 72 (56 –86)
Dengel, 2014 [85]	**CS** **W** (kg) = 57.2 ± 1.1 **BMI** (kg/m^2^) = 22.4 ± 0.4 **Leukemia** **W** (kg) = 55.4 ± 1.7 **BMI** (kg/m^2^) = 22.2 ± 0.5 **CNS** **W** (kg) = 58.1 ± 2.0 **BMI** (kg/m^2^) = 23.1 ± 0.6 **ST** **W** (kg) = 55.4 ± 1.4 **BMI** (kg/m^2^) = 21.8 ± 0.5 **HC** **W** (kg) = 57.1 ± 1.2 **BMI** (kg/m^2^) = 21.8 ± 0.4	**CS** **SBP** (mmHg) = 110.9 ± 1 **DBP** (mmHg) = 58.3 ± 0.7 **Leukemia** **SBP** (mmHg) = 110.8 ± 1.3 **DBP** (mmHg) = 58.6 ± 0.9 **CNS** **SBP** (mmHg) = 109.8 ± 1.3 **DBP** (mmHg) = 57.8 ± 0.9 **ST** **SBP** (mmHg) = 110.7 ± 1.4 **DBP** (mmHg) = 58.0 ± 1.1 **HC** **SBP** (mmHg) = 110.5 ± 1.0 **DBP** (mmHg) = 57.5 ± 0.7	N.A.	N.A.
Krystal, 2015 [86]	**CS** **W** (kg) = N.A. **BMI** (kg/m^2^) = 23.4 ± 5.4 **HC** **W** (kg) = N.A. **BMI** (kg/m^2^) = 23.7 ± 3.2	**CS** **SBP** (mmHg) = 114.3 ± 11.3 **DBP** (mmHg) = 72.2 ± 8.5 **HC** **SBP** (mmHg) = 114 ± 18.8 **DBP** (mmHg) = 69.4 ± 9.5	N.A.	N.A.
Okur, 2016 [87]	N.A.	N.A.	N.A.	N.A.
Masopustová, 2018 [88]	**CS** **W** (kg) = N.A. **BMI** (kg/m^2^) = 21.1 (19.3–25.1) **HC** **W** (kg) = N.A. **BMI** (kg/m^2^) = 19.97 (18.8–22.9)	**CS** **SBP** (mmHg) = 116 (105–121) **DBP** (mmHg) = 64 (60–70) **HC** **SBP** (mmHg) = 114.5 (110–119) **DBP** (mmHg) = 62.5 (59–69.5)	N.A.	**CS** **Total cholesterol** (mmol/L) = 4.2 (3.8–4.7) **HC** **Total cholesterol** (mmol/L) = 4.3 (3.9 –4.8)
Muggeo, 2019 [89]	**CS** **W** (kg) = N.A. **BMI** (SDS) = 0.9 ± 0.9 **CS 25-OHD < 20 ng/mL** **W** (kg) = N.A. **BMI** (SDS) = 0.9 ± 0.9 **CS 25-OHD > 20 ng/mL** **W** (kg) = N.A. **BMI** (SDS) = 0.89 ± 0.8 **HC** **W** (kg) = N.A. **BMI** (SDS) = 0.89 ± 0.8	N.A.	N.A.	**CS** = N.A. **CS 25-OHD < 20 ng/mL** **LDL-C** (mg/dL) = 87 ± 17 **HDL-C** (mg/dL) = 51 ± 10 **Total cholesterol** (mg/dL) = 152 ± 23 **Triglycerides** (mg/dL) = 70 ± 34 **CS 25-OHD > 20 ng/mL** **LDL-C** (mg/dL) = 86 ± 20 **HDL-C** (mg/dL) = 49 ± 9 controls **Total cholesterol** (mg/dL) = 149 ± 26 **Triglycerides** (mg/dL) = 66 ± 26 **HC** = N.A.
von Korn, 2019 [90]	**CS** **W** (kg) = 46.3 ± 18.3 **BMI** (z-score) = 0.21 ± 1.2 **HC** = N.A.	**CS** **SBP** (z-score) = 0.31 ± 1.1 **DBP** (z-score) = −0.30 ± 1.3 **HC** = N.A.	N.A.	N.A.
Keiser, 2020 [92]	**CS** **W** (kg) = 17.63 ± 3.26 **BMI** (kg/m^2^) = N.A.	N.A.	N.A.	N.A.
Sherief, 2021 [93]	**CS** **W** (kg) = 36.7 ± 9.5 **BMI** (kg/m^2^) = 18.04 ± 2.9 **HC** **W** (kg) = 36.10 ± 12.2 **BMI** (kg/m^2^) = 18.4 ± 4	**CS** **SBP** (mmHg) = 106 ± 6.2 **DBP** (mmHg) = 63.25 ± 5.1 **HC** **SBP** (mmHg) = 105 ± 5.1 **DBP** (mmHg) = 36.10 ± 3.7	N.A.	**CS** **LDL-C** (mg/dL) = 56.24 ± 25.7 **HDL-C** (mg/dL) = 58.88 ± 13.3 **Total cholesterol** (mg/dL) = 151.66 ± 15.7 **Triglycerides** (mg/dL) = 130.96 ± 10 HC **LDL-C** (mg/dL) = 56.24 ± 25.70 **HDL-C** (mg/dL) = 61.38 ± 10.8 **Total cholesterol** (mg/dL) = 124.87 ± 26.1 **Triglycerides** (mg/dL) = 120.79 ± 18.6

## Data Availability

All data is available in the manuscript.

## Abbreviations

AA	Anthracycline
aaIMT	Abdominal aorta intima-media thickness
AASI	Ambulatory arterial stiffness index
ABPM	Ambulatory blood pressure monitoring
AD	Adiponectin
ADMA	Asymmetric dimethylarginine
AEPC	Association for European Pediatric Cardiology
AHA	American Heart Association
AI	Augmentation index
ALL	Acute lymphoblastic leukemia
ALL-AP	Acute lymphocytic leukemia acute phase
ALL-CG	Acute lymphocytic leukemia control group
ALL-CR	Acute lymphocytic leukemia complete remission
AML	Acute myeloid leukemia
APAO	Antero posterior abdominal aorta diameter
APML	Acute promyelocytic leukemia
ARA-C	Cytosine arabinoside
ATG	Anti-T serum globulin
BAR	Brachial artery reactivity
BMI	Body mass index
BU	Busulphan
CB	Carboplatinum
CCS	Childhood cancer survivors
CD	Cumulative dose
CHT	Chemotherapy
cIMT	Carotid intima-media thickness
CMR	Cardiovascular magnetic resonance
CNS	Central nervous system
CRP	C-reactive protein
CRT	Cranial irradiation treatment
CS	Cancer survivors
CSBP	Central systolic blood pressure
CSD	Cross sectional distensibility
CT	Chemotherapy
CVD	Cardiovascular disease
CYC	Cyclophosphamide
DBP	Diastolic blood pressure
DD	Diameter distensibility
ECG	Electrocardiogram
EDD	Endothelial dependent dilation
EID	Endothelial-independent dilation
ES	Endothelial selectin
ESH	European Society of Hypertension
ESR	Erythrocyte sedimentation rate
ET-1	Endothelin-1
FG	Fasting glucose
FI	Fasting insulin
fIMT	Femoral artery intima-media thickness
FMD	Flow-mediated dilation
GY	Gray
HC	Healthy control
HDL	High-density lipoprotein
HGD	Hodgkin disease
HMW-AD	High-Molecular weight adiponectin
HRPF	Health-related physical fitness
hs-CRP	High sensitivity-C reactive protein
IFO	Isofosfamide
IL-6	Interleukin-6
LDL-C	Low-density lipoprotein cholesterol
LS	Leukocyte selectin
MAP	Mean arterial pressure
MPH	Melphalan
MPO	Myeloperoxidase
MRI	Magnetic resonance imaging
MS	Metabolic syndrome
MTX	Methotrexate
N.A.	Not available
NHL	Non-Hodgkin lymphoma
NO	Nitric oxide
NO_2_/NO_3_	Nitrate/nitrite
NTG	Nitrate-mediated dilatation
OR	Odds ratio
OX-LDL	Oxidized LDL
PA	Physical activity
PAT	Peripheral artery tonometry
PD	Platinum derivates
PDBP	Peripheral diastolic blood pressure
PDGF	Platelet-derived growth factor
PPao	Aortic pulse pressure
PSBP	Peripheral systolic blood pressure
PWA	Pulse wave analysis
PWV	Pulse wave velocity
PWVao	Aortic pulse wave velocity
PWVcf	Carotid-femoral pulse wave velocity
RHI	Reactive hyperemia index
ROS	Reactive oxygen species
RT	Radiotherapy
SBP	Systolic blood pressure
SBPao	Aortic systolic blood pressure
SCT	Stem cell transplantation
SI-B	Stiffness index beta
ST	Solid tumors
TAT	Thrombin antithrombin complex
TBI	Total body irradiation
TF	Tissue factor
TG	Triglycerides
TM	Thrombomodulin
TT	Thiotepa
VCAM	Vascular cell adhesive molecules
VEGF	Vascular endothelial growth factor
VOD	Veno-occlusive disease
vWF	von Willebrand factor
WBC	White blood cell
25-OHD	25-Hydroxyvitamin D

## References

[1] GBD 2015 Risk Factors Collaborators (2016). Global, regional, and national comparative risk assessment of 79 behavioural, environmental and occupational, and metabolic risks or clusters of risks, 1990–2015: A systematic analysis for the Global Burden of Disease Study 2015. Lancet.

[2] Armenian S.H., Armstrong G.T., Aune G., Chow E.J., Ehrhardt M.J., Ky B., Moslehi J., Mulrooney D.A., Nathan P.C., Ryan T.D. (2018). LCM. Cardiovascular Disease in Survivors of Childhood Cancer: Insights into Epidemiology, Pathophysiology, and Prevention. J. Clin. Oncol..

[3] Haupt R., Jankovic M., Hjorth L., Skinner R. (2013). Late effects in childhood cancer survivors and survivorship issues. Epidemiol. Prev..

[4] Meacham L.R., Chow E.J., Ness K.K., Kamdar K.Y., Chen Y., Yasui Y., Oeffinger K.C., Sklar C.A., Robison L.L., Mertens A.C. (2010). Cardiovascular risk factors in adult survivors of pediatric cancer—A report from the childhood cancer survivor study. Cancer Epidemiol. Biomark. Prev..

[5] Mertens A.C., Liu Q., Neglia J.P., Wasilewski K., Leisenring W., Armstrong G.T., Robison L.L., Yasui Y. (2008). Cause-specific late mortality among 5-year survivors of childhood cancer: The Childhood Cancer Survivor Study. J. Natl. Cancer Inst..

[6] Herrmann J. (2020). Vascular toxic effects of cancer therapies. Nat. Rev. Cardiol..

[7] Parr S.K., Liang J., Schadler K.L., Gilchrist S.C., Steele C.C., Ade C.J. (2020). Anticancer Therapy-Related Increases in Arterial Stiffness: A Systematic Review and Meta-Analysis. J. Am. Heart Assoc..

[8] Strongman H., Gadd S., Matthews A., Mansfield K.E., Stanway S., Lyon A.R., Dos-Santos-Silva I., Smeeth L., Bhaskaran K. (2019). Medium and long-term risks of specific cardiovascular diseases in survivors of 20 adult cancers: A population-based cohort study using multiple linked UK electronic health records databases. Lancet.

[9] Deanfield J., Donald A., Ferri C., Giannattasio C., Halcox J., Halligan S., Lerman A., Mancia G., Oliver J.J., Pessina A.C. (2005). Working Group on Endothelin and Endothelial Factors of the European Society of Hypertension. Endothelial function and dysfunction. Part I: Methodological issues for assessment in the different vascular beds: A statement by the Working Group on Endothelin and Endothelial Factors of the European Society of Hypertension. J. Hypertens..

[10] Van Hinsbergh V.W. (2012). Endothelium-role in regulation of coagulation and inflammation. Semin. Immunopathol..

[11] Franchi T., Eaton S., De Coppi P., Giuliani S. (2019). The emerging role of immunothrombosis in paediatric conditions. Pediatr. Res..

[12] Bellastella G., Scappaticcio L., Esposito K., Giugliano D., Maiorino M.I. (2018). Metabolic syndrome and cancer: “The common soil hypothesis”. Diabetes Res. Clin. Pract..

[13] Reaven G.M. (1988). Banting lecture 1988. Role of insulin resistance in human disease. Diabetes.

[14] Reisinger C., Nkeh-Chungag B.N., Fredriksen P.M., Goswami N. (2021). The prevalence of pediatric metabolic syndrome—A critical look on the discrepancies between definitions and its clinical importance. Int. J. Obes..

[15] Al-Hamad D., Raman V. (2017). Metabolic syndrome in children and adolescents. Transl. Pediatr..

[16] Ford E.S., Li C. (2008). Defining the metabolic syndrome in children and adolescents: Will the real definition please stand up?. J. Pediatr..

[17] Alberti K.G., Zimmet P., Shaw J. (2006). Metabolic syndrome—A new world-wide definition. A Consensus Statement from the International Diabetes Federation. Diabet. Med..

[18] Nuver J., Smit A.J., Wolffenbuttel B.H., Sluiter W.J., Hoekstra H.J., Sleijfer D.T., Gietema J.A. (2005). The metabolic syndrome and disturbances in hormone levels in long-term survivors of disseminated testicular cancer. J. Clin. Oncol..

[19] Talvensaari K.K., Lanning M., Tapanainen P., Knip M. (1996). Long-term survivors of childhood cancer have an increased risk of manifesting the metabolic syndrome. J. Clin. Endocrinol. Metab..

[20] Rogers P.C., Meacham L.R., Oeffinger K.C., Henry D.W., Lange B.J. (2005). Obesity in pediatric oncology. Pediatr. Blood Cancer.

[21] Ross J.A., Oeffinger K.C., Davies S.M., Mertens A.C., Langer E.K., Kiffmeyer W.R., Sklar C.A., Stovall M., Yasui Y., Robison L.L. (2004). Genetic variation in the leptin receptor gene and obesity in survivors of childhood acute lymphoblastic leukemia: A report from the Childhood Cancer Survivor Study. J. Clin. Oncol..

[22] Chueh H.W., Yoo J.H. (2017). Metabolic syndrome induced by anticancer treatment in childhood cancer survivors. Ann. Pediatr. Endocrinol. Metab..

[23] Kate A., Kadambari D. (2016). Incidence of metabolic syndrome in breast cancer survivors on adjuvant hormonal therapy. J. Pharmacol. Pharmacother..

[24] Darzy K.H., Shalet S.M. (2009). Hypopituitarism following radiotherapy. Pituitary.

[25] Tapio S., Little M.P., Kaiser J.C., Impens N., Hamada N., Georgakilas A.G., Simar D., Salomaa S. (2021). Ionizing radiation-induced circulatory and metabolic diseases. Environ. Int..

[26] Pluimakers V.G., van Waas M., Neggers S.J.C.M.M., van den Heuvel-Eibrink M.M. (2019). Metabolic syndrome as cardiovascular risk factor in childhood cancer survivors. Crit. Rev. Oncol. Hematol..

[27] Mayer E.I., Reuter M., Dopfer R.E., Ranke M.B. (2000). Energy expenditure, energy intake and prevalence of obesity after therapy for acute lymphoblastic leukemia during childhood. Horm. Res..

[28] Ness K.K., Hudson M.M., Ginsberg J.P., Nagarajan R., Kaste S.C., Marina N., Whitton J., Robison L.L., Gurney J.G. (2009). Physical performance limitations in the Childhood Cancer Survivor Study cohort. J. Clin. Oncol..

[29] Casco S., Soto-Vega E. (2016). Development of Metabolic Syndrome Associated to Cancer Therapy: Review. Horm. Cancer..

[30] Skrzypczyk P., Pańczyk-Tomaszewska M. (2017). Methods to evaluate arterial structure and function in children—State-of-the art knowledge. Adv. Med. Sci..

[31] Axtell A.L., Gomari F.A., Cooke J.P. (2010). Assessing endothelial vasodilator function with the Endo-PAT 2000. J. Vis. Exp..

[32] Bonetti P.O., Pumper G.M., Higano S.T., Holmes D.R., Kuvin J.T., Lerman A. (2004). Noninvasive identification of patients with early coronary atherosclerosis by assessment of digital reactive hyperemia. J. Am. Coll. Cardiol..

[33] Matsuzawa Y., Sugiyama S., Sugamura K., Nozaki T., Ohba K., Konishi M., Matsubara J., Sumida H., Kaikita K., Kojima S. (2010). Digital assessment of endothelial function and ischemic heart disease in women. J. Am. Coll. Cardiol..

[34] Kuvin J.T., Patel A.R., Sliney K.A., Pandian N.G., Sheffy J., Schnall R.P., Karas R.H., Udelson J.E. (2003). Assessment of peripheral vascular endothelial function with finger arterial pulse wave amplitude. Am. Heart J..

[35] Woo J.S., Jang W.S., Kim H.S., Lee J.H., Choi E.Y., Kim J.B., Kim W.S., Kim K.S., Kim W. (2014). Comparison of peripheral arterial tonometry and flow-mediated vasodilation for assessment of the severity and complexity of coronary artery disease. Coron. Artery Dis..

[36] Bhangoo A., Sinha S., Rosenbaum M., Shelov S., Ten S. (2011). Endothelial function as measured by peripheral arterial tonometry increases during pubertal advancement. Horm. Res. Paediatr..

[37] Radtke T., Khattab K., Eser P., Kriemler S., Saner H., Wilhelm M. (2012). Puberty and microvascular function in healthy children and adolescents. J. Pediatr..

[38] Selamet Tierney E.S., Newburger J.W., Gauvreau K., Geva J., Coogan E., Colan S.D., de Ferranti S.D. (2009). Endothelial pulse amplitude testing: Feasibility and reproducibility in adolescents. J. Pediatr..

[39] Järvisalo M.J., Rönnemaa T., Volanen I., Kaitosaari T., Kallio K., Hartiala J.J., Irjala K., Viikari J.S., Simell O., Raitakari O.T. (2002). Brachial artery dilatation responses in healthy children and adolescents. Am. J. Physiol. Heart Circ. Physiol..

[40] Leeson C.P., Whincup P.H., Cook D.G., Donald A.E., Papacosta O., Lucas A., Deanfield J.E. (1997). Flow-mediated dilation in 9- to 11-year-old children: The influence of intrauterine and childhood factors. Circulation.

[41] Urbina E.M., Williams R.V., Alpert B.S., Collins R.T., Daniels S.R., Hayman L., Jacobson M., Mahoney L., Mietus-Snyder M., Rocchini A. (2009). American Heart Association Atherosclerosis, Hypertension, and Obesity in Youth Committee of the Council on Cardiovascular Disease in the Young. Noninvasive assessment of subclinical atherosclerosis in children and adolescents: Recommendations for standard assessment for clinical research: A scientific statement from the American Heart Association. Hypertension.

[42] Hussein G., Bughdady Y., Kandil M.E., Bazaraa H.M., Taher H. (2008). Doppler assessment of brachial artery flow as a measure of endothelial dysfunction in pediatric chronic renal failure. Pediatr. Nephrol..

[43] Rahul I., Krishnamurthy S., Satheesh S., Biswal N., Bobby Z., Lakshminarayanan S. (2015). Brachial artery flow-mediated dilatation and carotid intima medial thickness in pediatric nephrotic syndrome: A cross-sectional case-control study. Clin. Exp. Nephrol..

[44] Järvisalo M.J., Raitakari M., Toikka J.O., Putto-Laurila A., Rontu R., Laine S., Lehtimäki T., Rönnemaa T., Viikari J., Raitakari O.T. (2004). Endothelial dysfunction and increased arterial intima-media thickness in children with type 1 diabetes. Circulation.

[45] Woo K.S., Chook P., Yu C.W., Sung R.Y., Qiao M., Leung S.S., Lam C.W., Metreweli C., Celermajer D.S. (2004). Overweight in children is associated with arterial endothelial dysfunction and intima-media thickening. Int. J. Obes. Relat. Metab. Disord..

[46] Woo K.S., Chook P., Yu C.W., Sung R.Y., Qiao M., Leung S.S., Lam C.W., Metreweli C., Celermajer D.S. (2004). Effects of diet and exercise on obesity-related vascular dysfunction in children. Circulation.

[47] Doyon A., Kracht D., Bayazit A.K., Deveci M., Duzova A., Krmar R.T., Litwin M., Niemirska A., Oguz B., Schmidt B.M. (2013). 4C Study Consortium. Carotid artery intima-media thickness and distensibility in children and adolescents: Reference values and role of body dimensions. Hypertension.

[48] Drole Torkar A., Plesnik E., Groselj U., Battelino T., Kotnik P. (2020). Carotid Intima-Media Thickness in Healthy Children and Adolescents: Normative Data and Systematic Literature Review. Front. Cardiovasc. Med..

[49] Dalla Pozza R., Ehringer-Schetitska D., Fritsch P., Jokinen E., Petropoulos A., Oberhoffer R., Association for European Paediatric Cardiology Working Group Cardiovascular Prevention (2015). Intima media thickness measurement in children: A statement from the Association for European Paediatric Cardiology (AEPC) Working Group on Cardiovascular Prevention endorsed by the Association for European Paediatric Cardiology. Atherosclerosis.

[50] Lurbe E., Agabiti-Rosei E., Cruickshank J.K., Dominiczak A., Erdine S., Hirth A., Invitti C., Litwin M., Mancia G., Pall D. (2016). 2016 European Society of Hypertension guidelines for the management of high blood pressure in children and adolescents. J. Hypertens..

[51] Dawson J.D., Sonka M., Blecha M.B., Lin W., Davis P.H. (2009). Risk factors associated with aortic and carotid intima-media thickness in adolescents and young adults: The Muscatine Offspring Study. J. Am. Coll. Cardiol..

[52] National High Blood Pressure Education Program Working Group on High Blood Pressure in Children and Adolescents (2004). The fourth report on the diagnosis, evaluation, and treatment of high blood pressure in children and adolescents. Pediatrics.

[53] Townsend R.R. (2017). Arterial Stiffness: Recommendations and Standardization. Pulse.

[54] Savant J.D., Furth S.L., Meyers K.E. (2014). Arterial Stiffness in Children: Pediatric Measurement and Considerations. Pulse.

[55] Martyn C.N., Greenwald S.E. (1997). Impaired synthesis of elastin in walls of aorta and large conduit arteries during early development as an initiating event in pathogenesis of systemic hypertension. Lancet.

[56] Tanaka H. (2018). Various Indices of Arterial Stiffness: Are They Closely Related or Distinctly Different?. Pulse.

[57] Laurent S., Cockcroft J., Van Bortel L., Boutouyrie P., Giannattasio C., Hayoz D., Pannier B., Vlachopoulos C., Wilkinson I., Struijker-Boudier H. (2006). Expert consensus document on arterial stiffness: Methodological issues and clinical applications. Eur. Heart J..

[58] Reusz G.S., Cseprekal O., Temmar M., Kis E., Cherif A.B., Thaleb A., Fekete A., Szabó A.J., Benetos A., Salvi P. (2010). Reference values of pulse wave velocity in healthy children and teenagers. Hypertension.

[59] Thurn D., Doyon A., Sözeri B., Bayazit A.K., Canpolat N., Duzova A., Querfeld U., Schmidt B.M., Schaefer F., Wühl E. (2015). 4C Study Consortium. Aortic Pulse Wave Velocity in Healthy Children and Adolescents: Reference Values for the Vicorder Device and Modifying Factors. Am. J. Hypertens..

[60] Elmenhorst J., Hulpke-Wette M., Barta C., Dalla Pozza R., Springer S., Oberhoffer R. (2015). Percentiles for central blood pressure and pulse wave velocity in children and adolescents recorded with an oscillometric device. Atherosclerosis.

[61] Salvi P., Furlanis G., Grillo A., Pini A., Salvi L., Marelli S., Rovina M., Moretti F., Gaetano R., Pintassilgo I. (2019). Unreliable Estimation of Aortic Pulse Wave Velocity Provided by the Mobil-O-Graph Algorithm-Based System in Marfan Syndrome. J. Am. Heart Assoc..

[62] Salvi P., Scalise F., Rovina M., Moretti F., Salvi L., Grillo A., Gao L., Baldi C., Faini A., Furlanis G. (2019). Noninvasive Estimation of Aortic Stiffness through Different Approaches. Hypertension.

[63] Hidvégi E.V., Jakab A.E., Lenkey Z., Bereczki C., Cziráki A., Illyés M. (2021). Updated and revised normal values of aortic pulse wave velocity in children and adolescents aged 3–18 years. J. Hum. Hypertens..

[64] Hidvégi E.V., Illyés M., Benczúr B., Böcskei R.M., Rátgéber L., Lenkey Z., Molnár F.T., Cziráki A. (2012). Reference values of aortic pulse wave velocity in a large healthy population aged between 3 and 18 years. J. Hypertens..

[65] Pereira T., Correia C., Cardoso J. (2015). Novel Methods for Pulse Wave Velocity Measurement. J. Med. Biol. Eng..

[66] Grotenhuis H.B., Westenberg J.J., Steendijk P., van der Geest R.J., Ottenkamp J., Bax J.J., Jukema J.W., de Roos A. (2009). Validation and reproducibility of aortic pulse wave velocity as assessed with velocity-encoded MRI. J. Magn. Reason. Imaging.

[67] Voges I., Jerosch-Herold M., Hedderich J., Pardun E., Hart C., Gabbert D.D., Hansen J.H., Petko C., Kramer H.H., Rickers C. (2012). Normal values of aortic dimensions, distensibility, and pulse wave velocity in children and young adults: A cross-sectional study. J. Cardiovasc. Magn. Reson..

[68] Stoner L., Faulkner J., Lowe A., Lambrick D.M., Young J.M., Love R., Rowlands D.S. (2014). Should the augmentation index be normalized to heart rate?. J. Atheroscler. Thromb..

[69] Li Y., Wang J.G., Dolan E., Gao P.J., Guo H.F., Nawrot T., Stanton A.V., Zhu D.L., O’Brien E., Staessen J.A. (2006). Ambulatory arterial stiffness index derived from 24-hour ambulatory blood pressure monitoring. Hypertension.

[70] Wühl E., Witte K., Soergel M., Mehls O., Schaefer F., German Working Group on Pediatric Hypertension (2002). Distribution of 24-h ambulatory blood pressure in children: Normalized reference values and role of body dimensions. J. Hypertens..

[71] Townsend R.R., Wilkinson I.B., Schiffrin E.L., Avolio A.P., Chirinos J.A., Cockcroft J.R., Heffernan K.S., Lakatta E.G., McEniery C.M., Mitchell G.F. (2015). American Heart Association Council on Hypertension. Recommendations for Improving and Standardizing Vascular Research on Arterial Stiffness: A Scientific Statement from the American Heart Association. Hypertension.

[72] Glowinska B., Urban M., Peczynska J., Florys B. (2005). Soluble adhesion molecules (sICAM-1, sVCAM-1) and selectins (sE selectin, sP selectin, sL selectin) levels in children and adolescents with obesity, hypertension, and diabetes. Metabolism.

[73] Budzyń M., Gryszczyńska B., Boruczkowski M., Kaczmarek M., Begier-Krasińska B., Osińska A., Bukowska A., Iskra M., Kasprzak M.P. (2019). The endothelial status reflected by circulating endothelial cells, circulating endothelial progenitor cells and soluble thrombomodulin in patients with mild and resistant hypertension. Vascul. Pharmacol..

[74] Vanhoutte P.M., Shimokawa H., Feletou M., Tang E.H. (2017). Endothelial dysfunction and vascular disease—A 30th anniversary update. Acta Physiol..

[75] Albiero M., Avogaro A., Fadini G.P. (2014). Circulating cellular players in vascular calcification. Curr. Pharm. Des..

[76] Zhang C. (2008). The role of inflammatory cytokines in endothelial dysfunction. Basic Res. Cardiol..

[77] Theofilis P., Sagris M., Oikonomou E., Antonopoulos A.S., Siasos G., Tsioufis C., Tousoulis D. (2021). Inflammatory Mechanisms Contributing to Endothelial Dysfunction. Biomedicines.

[78] Luzzatto G., Cella G., Messina C., Randi M.L., Sbarai A., Zanesco L. (2003). Markers of endothelial function in pediatric stem cell transplantation for acute leukemia. Med. Pediatr. Oncol..

[79] Chow A.Y., Chin C., Dahl G., Rosenthal D.N. (2006). Anthracyclines cause endothelial injury in pediatric cancer patients: A pilot study. J. Clin. Oncol..

[80] Hatzipantelis E.S., Athanassiou-Metaxa M., Gombakis N., Tzimouli V., Taparkou A., Sidi-Fragandrea V., Garipidou V., Papageorgiou T., Kleta D., Koliouskas D.E. (2011). Thrombomodulin and von Willebrand factor: Relation to endothelial dysfunction and disease outcome in children with acute lymphoblastic leukemia. Acta Haematol..

[81] Herceg-Cavrak V., Ahel V., Batinica M., Matec L., Kardos D. (2011). Increased arterial stiffness in children treated with anthracyclines for malignant disease. Coll. Antropol..

[82] Jang W.J., Choi D.Y., Jeon I.S. (2013). Vascular endothelial dysfunction after anthracyclines treatment in children with acute lymphoblastic leukemia. Korean J. Pediatr..

[83] Jenei Z., Bárdi E., Magyar M.T., Horváth A., Paragh G., Kiss C. (2013). Anthracycline causes impaired vascular endothelial function and aortic stiffness in long term survivors of childhood cancer. Pathol. Oncol. Res..

[84] Blair C.K., Kelly A.S., Steinberger J., Eberly L.E., Napurski C., Robien K., Neglia J.P., Mulrooney D.A., Ross J.A. (2014). Feasibility and preliminary efficacy of the effects of flavanoid-rich purple grape juice on the vascular health of childhood cancer survivors: A randomized, controlled crossover trial. Pediatr. Blood Cancer.

[85] Dengel D.R., Kelly A.S., Zhang L., Hodges J.S., Baker K.S., Steinberger J. (2014). Signs of early sub-clinical atherosclerosis in childhood cancer survivors. Pediatr. Blood Cancer.

[86] Krystal J.I., Reppucci M., Mayr T., Fish J.D., Sethna C. (2015). Arterial stiffness in childhood cancer survivors. Pediatr. Blood Cancer.

[87] Okur A., Karadeniz C., Özhan Oktar S., Pınarlı F.G., Aral A., Oğuz A. (2016). Assessment of brachial artery reactivity, carotid intima-media thickness, and adhesion molecules in pediatric solid tumor patients treated with anthracyclines. Pediatr. Hematol. Oncol..

[88] Masopustová A., Jehlička P., Huml M., Votava T., Trefil L., Kreslová M., Sýkora J. (2018). Plethysmographic and biochemical markers in the diagnosis of endothelial dysfunction in pediatric acute lymphoblastic leukemia survivors—New applications. Physiol. Res..

[89] Muggeo P., Muggeo V.M.R., Giordano P., Delvecchio M., Altomare M., Novielli C., Ciccone M.M., D’Amato G., Faienza M.F., Santoro N. (2019). Cardiovascular dysfunction and vitamin D status in childhood acute lymphoblastic leukemia survivors. World J. Pediatr..

[90] Von Korn P., Müller J., Quell C., Tenius L., Oberhoffer R., Feuchtinger T., Schmid I. (2019). Health-Related Physical Fitness and Arterial Stiffness in Childhood Cancer Survivors. Front. Cardiovasc. Med..

[91] Neuhauser H., Schienkiewitz A., Schaffrath Rosario A., Dortschy R., Kurth B.M. (2013). Referenzperzentile für Anthropometrische Maßzahlen und Blutdruck aus der Studie zur Gesundheit von Kindern und Jugendlichen in Deutschland (KiGGS).

[92] Keiser T., Gaser D., Peters C., Oberhoffer-Fritz R., Kesting S., von Luettichau I. (2020). Short-Term Consequences of Pediatric Anti-cancer Treatment Regarding Blood Pressure, Motor Performance, Physical Activity and Reintegration into Sports Structures. Front. Pediatr..

[93] Sherief L.M., Abd El-Khalek E.R., Libda I.A., Gaber O.A., Kamal N.M., Soliman B.K., Mokhtar W.A., Mokhtar G.A., Salah H.E., Kamar G.M. (2021). Serum endocan and endothelial dysfunction in childhood acute lymphoblastic leukemia survivors: A tertiary center experience. Ther. Adv. Chronic Dis..

[94] Chaosuwannakit N., D’Agostino R., Hamilton C.A., Lane K.S., Ntim W.O., Lawrence J., Melin S.A., Ellis L.R., Torti F.M., Little W.C. (2010). Aortic stiffness increases upon receipt of anthracycline chemotherapy. J. Clin. Oncol..

[95] Reference Values for Arterial Stiffness’ Collaboration (2010). Determinants of pulse wave velocity in healthy people and in the presence of cardiovascular risk factors: ‘Establishing normal and reference values’. Eur. Heart J..

[96] Feinstein S.B., Voci P., Pizzuto F. (2002). Noninvasive surrogate markers of atherosclerosis. Am. J. Cardiol..

[97] La Valle A., Crocco M., Chiarenza D.S., Maghnie M., d’Annunzio G. (2021). Endothelial impairment evaluation by peripheral arterial tonometry in pediatric endocrinopathies: A narrative review. World J. Diabetes.

[98] Poggesi A., Pasi M., Pescini F., Pantoni L., Inzitari D. (2016). Circulating biologic markers of endothelial dysfunction in cerebral small vessel disease: A review. J. Cereb. Blood Flow Metab..

[99] Szmitko P.E., Wang C.H., Weisel R.D., de Almeida J.R., Anderson T.J., Verma S. (2003). New markers of inflammation and endothelial cell activation: Part I. Circulation.

[100] Martínez-Miguel P., Valdivielso J.M., Medrano-Andrés D., Román-García P., Cano-Peñalver J.L., Rodríguez-Puyol M., Rodríguez-Puyol D., López-Ongil S. (2014). The active form of vitamin D, calcitriol, induces a complex dual upregulation of endothelin and nitric oxide in cultured endothelial cells. Am. J. Physiol. Endocrinol. Metab..

[101] Brouwer C.A., Postma A., Hooimeijer H.L., Smit A.J., Vonk J.M., van Roon A.M., van den Berg M.P., Dolsma W.V., Lefrandt J.D., Bink-Boelkens M.T. (2013). Endothelial damage in long-term survivors of childhood cancer. J. Clin. Oncol..

[102] Herrmann J. (2020). Adverse cardiac effects of cancer therapies: Cardiotoxicity and arrhythmia. Nat. Rev. Cardiol..

[103] Touyz R.M., Herrmann J. (2018). Cardiotoxicity with vascular endothelial growth factor inhibitor therapy. NPJ Precis. Oncol..

[104] Aktypis C., Spei M.E., Yavropoulou M., Wallin G., Koumarianou A., Kaltsas G., Kassi E., Daskalakis K. (2021). Cardiovascular Toxicities Secondary to Biotherapy and Molecular Targeted Therapies in Neuroendocrine Neoplasms: A Systematic Review and Meta-Analysis of Randomized Placebo-Controlled Trials. Cancers.

[105] Gebauer J., Higham C., Langer T., Denzer C., Brabant G. (2019). Long-Term Endocrine and Metabolic Consequences of Cancer Treatment: A Systematic Review. Endocr. Rev..

